# Spermidine/spermine-*N*^1^-acetyltransferase ablation impacts tauopathy-induced polyamine stress response

**DOI:** 10.1186/s13195-019-0507-y

**Published:** 2019-06-29

**Authors:** Leslie A. Sandusky-Beltran, Andrii Kovalenko, Chao Ma, John Ivan T. Calahatian, Devon S. Placides, Mallory D. Watler, Jerry B. Hunt, April L. Darling, Jeremy D. Baker, Laura J. Blair, Mackenzie D. Martin, Sarah N. Fontaine, Chad A. Dickey, April L. Lussier, Edwin J. Weeber, Maj-Linda B. Selenica, Kevin R. Nash, Marcia N. Gordon, Dave Morgan, Daniel C. Lee

**Affiliations:** 1 Byrd Alzheimer’s Institute, Department of Pharmaceutical Sciences, University of South Florida, 4001 E. Fletcher Ave, Tampa, FL 33613 USA; 2 Byrd Alzheimer’s Institute, Department of Molecular Medicine, University of South Florida, Tampa, FL 33613 USA; 3 Byrd Alzheimer’s Institute, Department of Molecular Pharmacology and Physiology, University of South Florida, Tampa, FL 33613 USA; 4 Neuroscience Institute, Department of Neuroscience and Physiology, New York University School of Medicine, 1 Park Avenue, New York, NY 10016 USA; 5 Department of Translational Science & Molecular Medicine, Michigan State University, 400 Monroe Ave NW, Grand Rapids, MI 49503 USA

**Keywords:** Tau, Polyamine dysregulation, Hippocampus, Alzheimer’s disease

## Abstract

**Background:**

Tau stabilizes microtubules; however, in Alzheimer’s disease (AD) and tauopathies, tau becomes hyperphosphorylated, aggregates, and results in neuronal death. Our group recently uncovered a unique interaction between polyamine metabolism and tau fate. Polyamines exert an array of physiological effects that support neuronal function and cognitive processing. Specific stimuli can elicit a polyamine stress response (PSR), resulting in altered central polyamine homeostasis. Evidence suggests that elevations in polyamines following a short-term stressor are beneficial; however, persistent stress and subsequent PSR activation may lead to maladaptive polyamine dysregulation, which is observed in AD, and may contribute to neuropathology and disease progression.

**Methods:**

Male and female mice harboring tau *P301L* mutation (rTg4510) were examined for a tau-induced central polyamine stress response (tau-PSR). The direct effect of tau-PSR byproducts on tau fibrillization and oligomerization were measured using a thioflavin T assay and a N2a split superfolder GFP-Tau (N2a-ssGT) cell line, respectively. To therapeutically target the tau-PSR, we bilaterally injected caspase 3-cleaved tau truncated at aspartate 421 (AAV9 Tau ΔD421) into the hippocampus and cortex of spermidine/spermine-*N*^1^-acetyltransferase (SSAT), a key regulator of the tau-PSR, knock out (SSAT-/-), and wild type littermates, and the effects on tau neuropathology, polyamine dysregulation, and behavior were measured. Lastly, cellular models were employed to further examine how SSAT repression impacted tau biology.

**Results:**

Tau induced a unique tau-PSR signature in rTg4510 mice, notably in the accumulation of acetylated spermidine. In vitro, higher-order polyamines prevented tau fibrillization but acetylated spermidine failed to mimic this effect and even promoted fibrillization and oligomerization. AAV9 Tau ΔD421 also elicited a unique tau-PSR in vivo, and targeted disruption of SSAT prevented the accumulation of acetylated polyamines and impacted several tau phospho-epitopes. Interestingly, SSAT knockout mice presented with altered behavior in the rotarod task, the elevated plus maze, and marble burying task, thus highlighting the impact of polyamine homeostasis within the brain.

**Conclusion:**

These data represent a novel paradigm linking tau pathology and polyamine dysfunction and that targeting specific arms within the polyamine pathway may serve as new targets to mitigate certain components of the tau phenotype.

**Electronic supplementary material:**

The online version of this article (10.1186/s13195-019-0507-y) contains supplementary material, which is available to authorized users.

## Background

Alzheimer’s disease (AD) is a progressive neurodegenerative process that affects distinct neurons and circuits that control memory and cognitive functions. Although tau is intrinsically disordered, under normal conditions, it stabilizes microtubules in neurons; however, in AD and other tauopathies, tau undergoes several post translational modifications, including abnormal phosphorylation, oxidation, conformational changes, and truncation, all which have been reported to contribute to its aggregation into paired helical filaments (PHFs), which make up neurofibrillary tangles (NFTs) [1, 2]. Importantly, tau accumulation remains the closest corollary to neuronal loss and cognitive decline in AD [3–7]. Recent studies have identified tau cleavage by caspase activation at aspartate 421 (tau ΔD421) as an early event in the pathologic cascade leading to NFT formation [8, 9], a phenomena that has been observed in AD [9–12] and other tauopathies [13–15] and may contribute to pathogenesis and neurotoxicity [16].

**Fig. 1:**
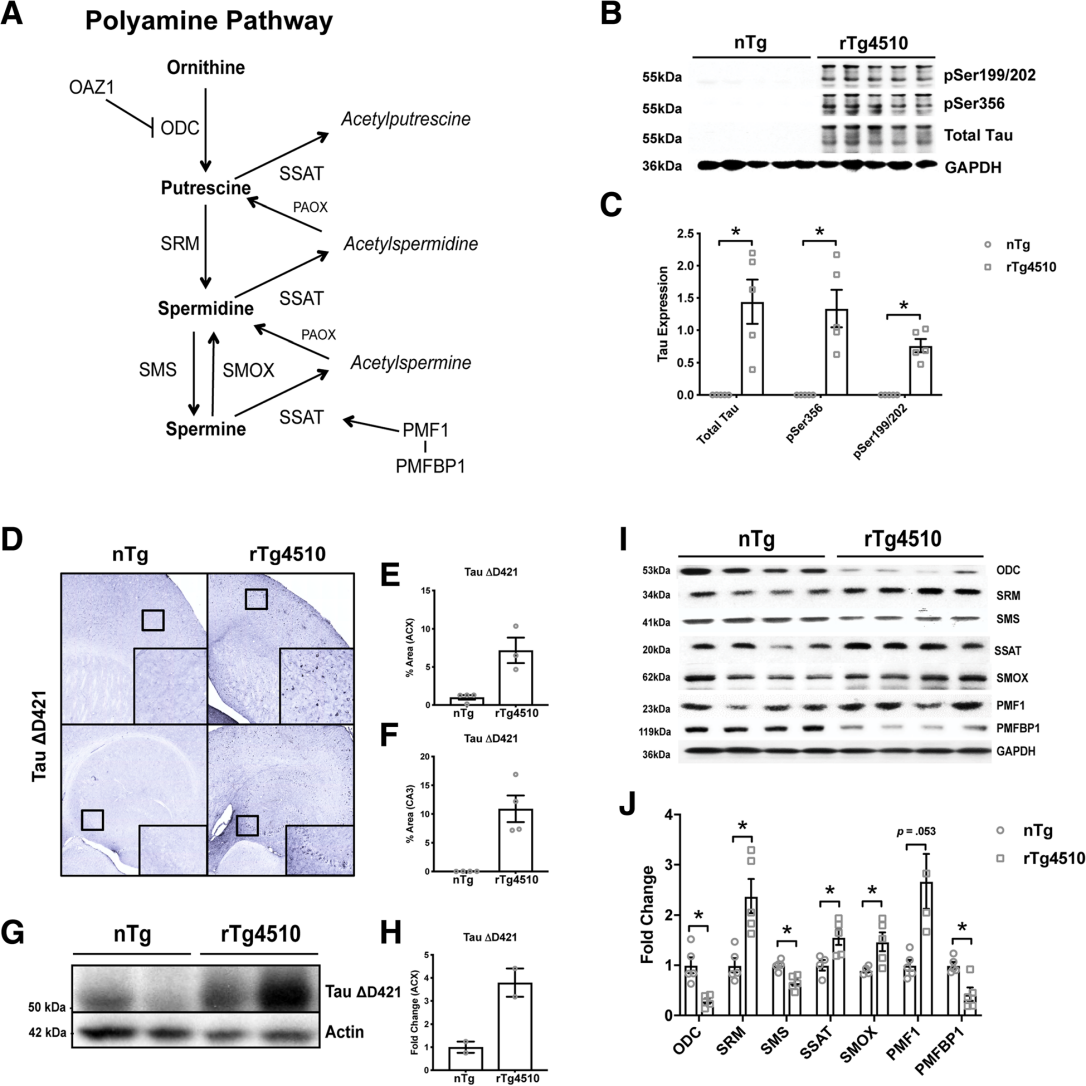
Tau rTg4510 mice show significant polyamine dysregulation. **a** Simplified polyamine pathway. Ornithine decarboxylase antizyme 1 (OAZ1), ornithine decarboxylase (ODC), spermidine synthase (SRM), spermidine synthase (SMS), spermine oxidase (SMOX), spermidine/spermine-*N*^1^-acetyltransferase (SSAT), polyamine oxidase (PAOX), polyamine-modulated factor 1 (PMF1), polyamine modulated factor binding protein 1 (PMFBP1). **b**, **c** Representative images and quantification of western blot analysis of 12-month-old nTg and rTg4510 (*n* = 5) hippocampal tau neuropathology (pSer199/202: *t* (4^+^) = − 7.490, *p* = .002; pSer356: *t* (4^+^) = − 4.588, *p* = .010; and total tau (H150): *t* (4^+^) = − 4.201, *p* = .014), followed by quantification (*n* = 5). Independent sample *t* test with ^+^Levene’s test for equality of variance correction, *p < .05. Data is represented by means ± S.E.M. **d**–**h** Representative images and quantification of immunohistochemical and western blot analysis of 14-month-old nTg and rTg4510 (*n* = 2–4) cortical and hippocampal C-terminally truncated tau neuropathology (tau ΔD421). Data is represented by means ± S.E.M. **i**–**j** Representative images and quantification of western blot analysis of 12-month-old nTg and rTg4510 (*n* = 4–5) polyamine dysregulation (ODC: *t* (4.843^+^) = 4.320, *p* = .008; SRM: *t* (5.347^+^) = − 3.756, *p* = .012; SMS: *t*(8) = 4.412, *p* = .003; SSAT: *t*(7) = − 2.849, *p* = .025; SMOX: *t* (4.399^+^) = − 2.958, *p* = .037; PMF1: *t* (4.113^+^) = − 2.690, *p* = .053; PMFBP1: *t*(8) = 3.850, *p* = .005). Independent sample *t* test with ^+^Levene’s test for equality of variance correction, *p < .05. Data is represented by means ± S.E.M.

Polyamines exert an array of physiological effects that support neuronal function and axonal integrity [17–21]. The biosynthesis of polyamines remains under the control of key regulatory enzymes (see Fig. 1a for simplified pathway). One enzyme in particular, spermidine/spermine-*N*^1^-acetyltransferase (SSAT), together with polyamine oxidase (PAOX) or spermine oxidase (SMOX), produces the epiphenomenon known as the “polyamine back-conversion cascade” to recycle higher-order polyamines when polyamine levels increase. Through this recycling process, SSAT acetylates polyamines creating putatively inactive acetylated forms. The acetylated forms can either convert to intermediate or lower-order polyamines or persist as acetylated polyamines resulting in efflux from the cell [22]. This system remains tightly controlled to regulate the levels of polyamines. Polyamines possess cognitive modulating properties [23–29]; therefore, dysregulation in this system may impact normal cognitive function.

Physical and emotional stimuli are known to elicit a “polyamine stress response” (PSR), resulting in altered central polyamine homeostasis [30–32]. The PSR remains a common adaptive response to stressful stimuli that can vary in magnitude, intensity, and recurrence. Evidence suggests that while the elevations in polyamines following a short-term stressor are beneficial, persistent stress and subsequent PSR activation may become maladaptive and result in chronic or long-term polyamine dysregulation. Despite being relatively unexplored in the field of neurodegeneration, polyamine dysregulation occurs in tauopathies [20, 33, 34], including AD [35–42], and may contribute to pathology and cognitive impairments. We propose pathological tau accumulation as a chronic physiological stressor that is capable of eliciting a unique tau-induced PSR (which we refer to as a tau-PSR).

Herein, we demonstrate that tau induces a polyamine stress response (tau-PSR) in two models harboring tau neuropathology. We show that higher-order polyamines prevent tau fibrillization, but the acetylated spermidine failed to mimic this affect and even promoted fibrillization. Further, targeted disruption of SSAT prevented the tau-PSR and partial accumulation of monomeric/high molecular weight tau multimers but also modulated behavior. Our data highlight the impact of tau-induced polyamine dysregulation and may provide alternative strategies to rebalance polyamine homeostasis and mitigate certain components of the tau phenotype.

## Methods

### Mice

All animal procedures were performed in accordance with the Institutional Animal Care and Use Committee (IACUC) at University of South Florida Health Byrd Alzheimer’s Institute. Male and female rTg4510 mice [43] were used for western blot (aged 12 months) and polyamine quantification (aged 8 months) experiments and have been previously described [44]. For biochemical and polyamine quantification experiments group sample sizes and sex distribution were as follows: nTg: *n* = 7 (1M/6F); rTg4510 *n* = 8 (4M/4F). For biochemical analyses, a random selection of *n* = 5 per group were used. For immunohistochemical experiment group sample sizes and sex distribution were as follows: nTg: *n* = 2 (0M/2F); rTg4510 *n* = 2 (1M/1F). For all experiments, statistical outliers were defined as falling more than two standard deviations from the mean and were removed prior to analyses (see “Statistical analyses” section).

Male and female spermidine/spermine *N*^1^-acetyltransferase (SSAT) null mice (SSAT-/-) on the C57BL/6 background were used for all other experiments (aged 11 months at time of surgery) and were provided as a generous gift from collaborator M. Soleimani at University of Cincinnati College of Medicine, Cincinnati, OH, USA. SSAT-/- mice comprise of a conventional disruption (neo cassette insertion in the SAT1 gene), thereby affecting all cell types that govern SSAT regulation and show altered polyamine homeostasis [45, 46]. SSAT transcript expression was confirmed by RT-PCR with primers targeting exon 1-3 (Fig. 7g). For immunohistochemical, biochemical, polyamine quantification, and behavioral experiments, group sample sizes and sex distribution were as follows: nTg AAV9 empty capsid: *n* = 10 (3M/7F); nTg AAV9 Tau ΔD421: *n* = 11 (8M/3F); SSAT-/- AAV9 empty capsid: *n* = 11 (7M/4F); SSAT-/- AAV9 Tau ΔD421: *n* = 10 (7M/3F). For biochemical analyses, a random selection of *n* = 5 per group were used. For electrophysiology analyses, group sample sizes and sex distribution were as follows [1]: nTg: *n* = 6 (6M/0F) [2]; SSAT-/-: *n* = 6 (6M/0F). For all experiments, statistical outliers were defined as falling more than two standard deviations from the mean and were removed prior to analyses (see “Statistical analyses” section).

### Thioflavin T assay

Recombinant 4R0N (P301L) tau was purified as described [47]. For the thioflavin T (ThT) assay, 10 μM tau was combined with 10 μM ThT (Sigma-Aldrich, St. Louis, MO, USA) in 10-mM sodium phosphate, pH 7.4 buffer. Compounds at 300 μM, putrescine (Sigma-Aldrich, St. Louis, MO, USA), spermidine (Sigma-Aldrich, St. Louis, MO, USA), spermine (Sigma-Aldrich, St. Louis, MO, USA), acetylputrescine (Sigma-Aldrich, St. Louis, MO, USA), acetylspermidine (Sigma-Aldrich, St. Louis, MO, USA), acetylspermine (Sigma-Aldrich, St. Louis, MO, USA), or vehicle control were added to as indicated in a black, clear bottom 96-well plate (Nunc). Compounds and vehicle controls were kept strictly to 5% of the total sample volume. Aggregation was initiated with 4 μM heparin, less than 2% of total well volume, and samples were incubated in a BioTek Synergy H1 plate reader for 10-min readings for a 72-h period at excitation wavelength 442 nm and emission wavelength 482 nm. Each compound was assessed in triplicate wells and compared to tau + vehicle. Average Buffer background was subtracted and accounted for at each time point. Lowest tau at 0 h time point was created, as it was lowest tau relative fluorescence unit (RFU). That RFU value of tau was used and subtracted from each compound at each time point (RFU value of tau used to subtract was from the 0 h.). Hours were calculated by doing a 1/6, 2/6… 433/6 to calculate hours from 10-min readings that were taken.

### Split GFP-Tau oligomerization assay

The split GFP-Tau plasmids pmGFP10C-Tau (#71433) and pmGFP11C-Tau (#71434) were purchased from Addgene and used together to visualize tau dimerization [48, 49]. These two plasmids were desired for bimolecular fluorescence complementation (BiFC) experiment based on the complementary superfolder GFP split into β-strands 1–10 (1–212 aa) and the 11th β-strand (213–228 aa). The GFP fragments were fused to the C-terminus of tau (0N4R). Both plasmids were sequenced to confirm their correct identity. Neuro-2a cells (N2a, mouse neuroblastoma, ATCC® CCL131™) were cultured in DMEM supplemented with 10% fetal bovine serum, 1% (*v*/*v*) GlutaMAX (2 mM), penicillin (100 IU/ml), streptomycin (100 μg/ml), and sodium pyruvate (1 mM) at 37 °C in a 5% CO_2_ humidified incubator. For functional validation of the two split GFP-Tau plasmids, both were individually and co-transfected into N2a cells using Lipofectamine 2000 (Life Technologies) according to manufacturer’s protocols. Twenty-four hours post transfection, cells were imaged at 4× and 20× under bright field and GFP fluorescence by using Cytation™ 3 Cell Imaging Multi-Mode Reader (BioTek™). 72-hours post transfection, cells co-transfected with both plasmids were selected with zeocin (125 μg/ml, R25001, Life Technologies) and geneticin (400 μg/ml, 30234CI, Corning) in N2a complete medium for obtaining stable transfected clones. After a total of 6-month selection process, several single stable clones were picked and eventually proliferated into stable monoclonal cell lines. The novel cell line named N2a split superfolder GFP-Tau (N2a-ssGT) was established and characterized by detecting high percentage of GFP using Accuri® C6 flow cytometer (BD Biosciences) under FL1 detector (Laser 488). Thus, N2a-ssGT cells were used as a model for visualizing tau oligomerization. For split GFP-Tau oligomerization assay, N2a-ssGT cells were plated on 96-well assay plate (3603, Corning), then treated with *N*^8^-acetylspermidine dihydrochloride (A3658, Sigma-Aldrich) at a final concentration of 30 μM on the following day. The medium for diluting the drug was supplemented with 1 mM aminoguanidine hydrochloride (396494-25G, Sigma-Aldrich) to inhibit bovine amine oxidases activity in the fetal bovine serum. Twenty-four, 48, and 72 h post treatment, the 96-well plate was automatically read to collect GFP fluorescence arbitrary units (A.U.) and imaged simultaneously under an optimized preset protocol on the Cytation™ 3 reader.

### Viral production

Truncated human tau 1-421 amino acids (tau ΔD421) were cloned into the recombinant adeno-associated virus (rAAV) vector pTR2-MCS. This vector expresses the tau with the chicken beta-actin CMV hybrid promoter (CBA) and contains the AAV2 terminal repeats. The tau ΔD421 was also appended on the N-terminus with a hemagglutinin (HA) tag for easier detection of the expressed protein. rAAV serotype 9 viral particles were generated from a triple transfection into HEK293 cells, followed by purification as described previously [50]. Viral titer was performed via dot blot as described previously [51].

### Surgical procedures

At 11 months of age, SSAT null mice and SSAT sufficient littermates were anesthetized by isoflurane, and a volume of 2 μL of rAAV particles containing rAVV9-empty capsid or C-terminally truncated tau (tau ΔD421) (3 × 10^12^ vg/ml) were stereotaxically injected (David Kopf Instruments, Tujunga, CA, USA) bilaterally into the CA3 of the hippocampus and the anterior cortex. Stereotaxic coordinates from bregma were AP = − 2.5 mm, ML = ± 2.9 mm, DV = − 3.0 mm, for the hippocampus, and AP = 2.2 mm, ML = ± 1.7 mm, and DV = − 3.0 for the cortex. Virus was administered using convection-enhanced delivery at a constant rate of 1.5 μL/min [52]. Viral incubation was 3 months at time of behavioral testing and 4 months at time of euthanasia and tissue collection.

### Behavioral testing

Three months after viral incubation, mice performed a battery of behavioral tasks to assess alterations in cognitive performance, motor performance, and anxiety. Behavioral tasks were run in order of increasing stress to minimize stress-effects on behavioral performance. White noise (55 dB) was present during all testing.

#### Open field maze

The open field (OF) apparatus (Ugo Basile, Italy) is a large open area that measures in centimeters: 44 L × 44 W × 30 H. Each mouse was allowed to freely explore the apparatus for 5 min. Total distance traveled, number of entries to the center zone, and time in the center zone was calculated by AnyMaze software (Stoelting Company, Wood Dale, IL) to measure anxiety-like behavior.

#### Elevated plus maze

The elevated plus maze (EPM) apparatus (Ugo Basile, Italy) is a “+”-shaped maze with two facing open arms, two facing closed arms, and a center area (arm/wall sizes, in centimeters, 35 L × 5 W × 15 H; runway height from floor, 50 cm). Each mouse was allowed to freely explore the maze for 5 min. The number of entries into the open arms, and time spent in the open arms, was calculated by AnyMaze software (Stoelting Company, Wood Dale, IL) to measure anxiety-like behavior and behavioral disinhibition.

#### Marble burying task

Rat breeding cages (in centimeters, 45 L × 20 W × 20 H) were filled with fresh pine bedding (Petspick), followed by gently overlaying 15 pink and white glass marbles (in centimeters, 1.25 diameter) equidistant in a 3 × 5 arrangement. Each mouse was allowed to explore for 30 min. Percentage of marbles buried (MB) (> 50% marble covered by bedding material) was blindly calculated as a measure of compulsion.

#### Rotarod

Mice were placed on the 3 cm diameter Rotarod (Ugo Basile, Italy) at an initial rotation speed of 4 rpm and accelerated to 40 rpm over 5 min. Mice performed four trials for two consecutive days and latency to fall was blindly recorded to measure motor performance and motor memory.

#### Radial arm water maze

Radial arm water maze was performed as previously described [53], with minor modifications. Briefly, the radial arm water maze (RAWM) contains six swim paths (arms) extending out of an open central area, with an escape platform located at the end of one arm (the goal arm). Visual cues are located on the walls surrounding the maze. The goal arm location remains constant for a given mouse, while the start arm is randomized for each trial. An average cohort size of *n* = 10 mice was used and all mice in a given cohort were run through each trial in sequential order. On day 1, mice are trained for 12 trials, with trials alternating between visible and hidden platforms. On day 2, mice are trained for 12 trials with only the hidden platform. Entry into an incorrect arm is scored as an error. The number of errors for two trials (hidden and visible) is averaged into blocks (B1–B6) across both days of testing, with number of errors being described as a measure of spatial working memory. Performance on day 1 can be further characterized as new learning, while performance on day 2 can be characterized as recognition and/or retrieval of a previously stored memory (acquired on day 1).

### Immunohistochemistry and Staining (IHC)

Following the completion of the one-month long behavioral testing battery, mice were euthanized with SomnaSol and transcardially perfused with 0.9% saline at 15 months of age, with a total of 4 months of viral incubation. One hemisphere of the brain was dissected, frozen, and stored at – 80 °C for biochemical analysis; the opposite hemisphere was fixed in 4% paraformaldehyde in 100 mM phosphate buffer (pH 7.4) for 24 h. Tissue was cryoprotected by sequential immersion in 10%, 20%, and 30% sucrose solutions. Brains were sectioned at 25 μm using a sliding microtome and stored in 4 °C in Dulbecco’s phosphate-buffered saline (DPBS) containing 100 mM sodium azide until staining.

Immunohistochemistry was performed on free-floating sections (6–8 per mouse) as previously described [54]. Antibodies were as follows: rabbit anti-Iba1 (1:3000; Wako Chemicals USA, Inc.), mouse anti-NeuN biotin (1:30,000; EMD Millipore), mouse anti-HT7 biotin (1:5000, Thermo Fisher Scientific), rabbit anti-Tau pS199/202 (1:5000; AnaSpec), and mouse paired helical filament tau biotin (AT8, 1:5000, Thermo Fisher Scientific).

### Biochemical analysis/western blotting

For the in vivo samples, dissected hippocampal tissue was used for western blot (WB) analysis and prepared as previously described [44] or using an AllPrep DNA/RNA/Protein Mini Kit (Qiagen, Germantown, MD), according to the manufacturer’s protocol. The detergent-soluble fraction (S1/SF) was subjected to western blot analysis. Protein concentration was determined with the Pierce BCA protein assay. Protein was loaded onto a 4–20% gradient gel (20- to 26-well Midi SureLock System; Life Technologies). Antibodies were as follows: mouse anti-Tau C3 (tau ΔD421)(1:1000; Sigma Aldrich), mouse anti-HT7 (1:1000, Thermo Fisher Scientific), mouse anti-Tau-5 (1:1000, Thermo Fisher Scientific), rabbit pS199/202 (1:1000; AnaSpec), mouse anti-Tau PHF (AT8; 1:1000; Thermo Fisher Scientific), rabbit anti-Tau pS396 (1:1000; AnaSpec), rabbit anti-ODC (1:1000; Epitomics), rabbit anti-OAZ1 (1:1000; Pierce), rabbit anti-SRM (1:1000; Epitomics), rabbit anti-SMS (1:1000; Proteintech), rabbit anti-SMOX (1:1000; Proteintech Group), rabbit anti-PMF1 (1:1000; Proteintech Group), rabbit anti-PMFBP1 (1:1000; Proteintech Group), and mouse anti-GAPDH (1:800,000; Meridian).

### Polyamine quantification

Polyamine quantification in brain homogenate was performed in collaboration with Sanford Burnham Prebys (Orlando, FL, USA) by liquid chromatography tandem mass spectrometry (LC-MS/MS) using a standard curve of each polyamine and acetylated polyamine (Sigma Aldrich).

### qRT-PCR

Total RNA from each mouse hippocampus was extracted using Qiagen’s All Prep DNA/RNA/Protein kit and used for RT-qPCR. A standard curve spanning two logs of a dynamic range was generated from a pool of naïve mice and experimental groups. Fifty nanograms of RNA from each mouse were used to generate cDNA using Superscript II First Strand Synthesis System (Life Technologies). Integrated DNA Technologies (IDT) PrimeTime® qPCR gene primers were used (SAT1: Mm.PT.58.45831345; GAPDH: Mm.PT.39a.1) with SYBR green-based real-time qPCR (Sigma-Aldrich). Quantitative PCR reaction proceeded by the following protocol: incubation at 95 °C/5 min, followed by 39 cycles of incubation at 95 °C/30 s, amplification at 60 °C/1 min, and a plate read. Primers were validated after each cycle by performing a dissociation curve analysis. The DNA Engine Opticon 2 TM Real-Time PCR System (Version 4.3; Bio-Rad) was used to detect the amplicon.

### Electrophysiology

Hippocampal slice electrophysiology was performed on 7-month-old SSAT-/- and non-transgenic littermates as previously described [54–56]. Briefly, baseline stimulus intensity was set at the level that elicited 40–50% of the maximum fEPSP response as determined from the input-output curve. Input-output relationships were determined by stimulating slices from 1–15 at 0.5 mV increments. Short-term plasticity was measured via paired-pulse facilitation, which was induced by stimulating slices at half-max intensity with sequential pulses spaced at 20 ms intervals from 20–300 ms. Long-term potentiation (LTP) was induced by a theta-burst protocol, which consisted of five bursts at 200 Hz separated by 200 ms, repeated six times with an inter-train interval of 10 s as previously described [56–58].

### siRNA knockdown of SAT1 in C3 HeLa cell culture

HeLa cells stably overexpressing 4R0N wild-type human tau (C3 HeLa) were used to model tau biology. Cells were maintained in 75 cm^2^ flasks at 37 °C in a 5% CO_2_ humidified chamber in OPTIMEM containing 10% fetal bovine serum, 100 U/ml penicillin and 100 ug/ml streptomycin. C3 HeLa cells were transfected with scramble negative siRNA control or SAT1 (FlexiTube siRNA; negative control siRNA, Hs_SAT_5 FlexiTube siRNA, Qiagen) using DhamaFECT1 (Horizon, Dharmacon) with or without 10 uM N1, N11-diethylnorspermine (DENSPM/DSPM; Tocris Cat#0468 batch NO. 4, Minneapolis, MN) (used to induce SAT1) for 72 h. Cells were harvested 72 h post transfection and whole cell pellets were lysed using M-PER (Thermo Scientific). Protein concentration was determined using Pierce BCA protein Assay (Thermo Scientific) and subjected to western blot analysis. Antibodies were as follows: rabbit anti-Tau pSer356 (1:1000; AnaSpec), rabbit anti-Tau pSer396 (1:1000, AnaSpec), mouse total tau (H150 (discontinuted); 1:1000; SantaCruz), rabbit anti-SAT1 (SSAT; 1:1000, Abcam), and mouse anti-GAPDH (1:800,000; Meridian).

### Statistical analyses

All statistical analyses were performed using SPSS Version 24. An independent samples *t* test was used for all non-transgenic (nTg) and rTg4510 comparisons, with a Levene’s test for equality of variances correction when appropriate. Data is represented by means ± S.E.M. *p < .05. Area under the curve (AUC) was calculated using GraphPad Prism, and data is represented as averages of triplicate wells for thioflavin T assays. A 2 × 3 factorial analysis of variance (ANOVA) (treatment × time) was used to determine the simple main effects of drug (vehicle vs 30 μM acetylspermidine) and time (24 h vs 48 h vs 72 h) as well as interaction of variables, followed by post hoc comparisons to further investigate specific effects of time within each drug on tau oligomerization. A 2 × 2 factorial analysis of variance (ANOVA) (genotype × viral treatment) was used to determine simple main effects of genotype (nTg vs SSAT-/-) and treatment (AAV9 empty capsid vs AAV9 Tau ΔD421) as well as interaction of variables, followed by pair-wise comparisons to further investigate specific effects of treatment within each genotype. A repeated measures mixed ANOVA was performed to determine simple main effects of genotype (nTg vs SSAT-/-) and treatment (AAV9 empty capsid vs AAV9 Tau ΔD421) as well as interaction of variables on performance across blocks in the radial arm water maze and trials in the rotarod task. A nonlinear regression was performed, and the line fits were compared using the extra sum-of-squares *F* test for electrophysiology experiments. A one-way analysis of variance (ANOVA) followed by Fisher’s PLSD post hoc comparisons was used to determine the effect of treatment in the siRNA cell culture experiments. For all analyses, statistical outliers were defined as falling more than two standard deviations from the mean. Data is represented by means ± S.E.M., *p* < .05; asterisks indicate the main effect of treatment and main effect of genotype, ampersands indicate the interaction of genotype and treatment, and number signs indicate the pairwise comparison within genotype.

## Results

### Tau rTg4510 mice show significantly disrupted polyamine homeostasis

Tau rTg4510 harbor the *MAPT P301L* mutation on a tetracycline response element regulated by the calcium/calmodulin-dependent protein kinase type II alpha chain (CaMKII-α) promoter. Mice deposit tau, phospho-tau (Fig. 1b, c), and tangles (not shown) in the forebrain and hippocampus but also develop many components of tauopathies including inflammation and atrophy [43, 44, 59]. One prominent post translational modification that incites further tau deposition and neuropathology includes cleavage by caspase-3 at aspartate 421 (tau ΔD421) which associates with Pick’s disease (PiD) [15], corticobasal degeneration (CBD) [60], progressive supranuclear palsy (PSP) [14], and AD [9–12]. Further, tau ΔD421 has been shown to increase tau spreading [61] and produce cognitive impairment [62].

We show tau ΔD421 in rTg4510 mice (Fig. 1d–h), which was associated with polyamine dysregulation at the level of enzymatic and protein control (Fig. 1i, j). Specifically, pro-synthetic enzymes ornithine decarboxylase (ODC), and spermine synthase (SMS) significantly decreased, whereas catabolic enzymes, spermidine/spermine-*N*^1^-acetyltransferase (SSAT) and spermine oxidase (SMOX) significantly increased. Spermidine synthase (SRM) also significantly increased, possibly as a compensatory mechanism in response to reduced ODC. Furthermore, polyamine-modulated factor1 (PMF1; a transcription factor that binds NF-E2 released factor 2 (Nrf-2) to increase SSAT expression [63]) also moderately increased (Fig. 1i, j), relative to non-transgenic controls. Importantly, the reduction in ODC and increase in SSAT were most notable, as they alone are thought to be the key regulatory enzymes to control polyamine homeostasis in the brain and provide evidence for a unique tau-PSR [30]. Although the role of polyamine-modulated factor 1 binding protein 1 (PMFBP1) is unknown, expression significantly decreased in tau transgenic mice. These data indicate that the tau phenotype renders dysregulation of anabolic and catabolic enzymes throughout the polyamine axis.

**Fig. 2:**
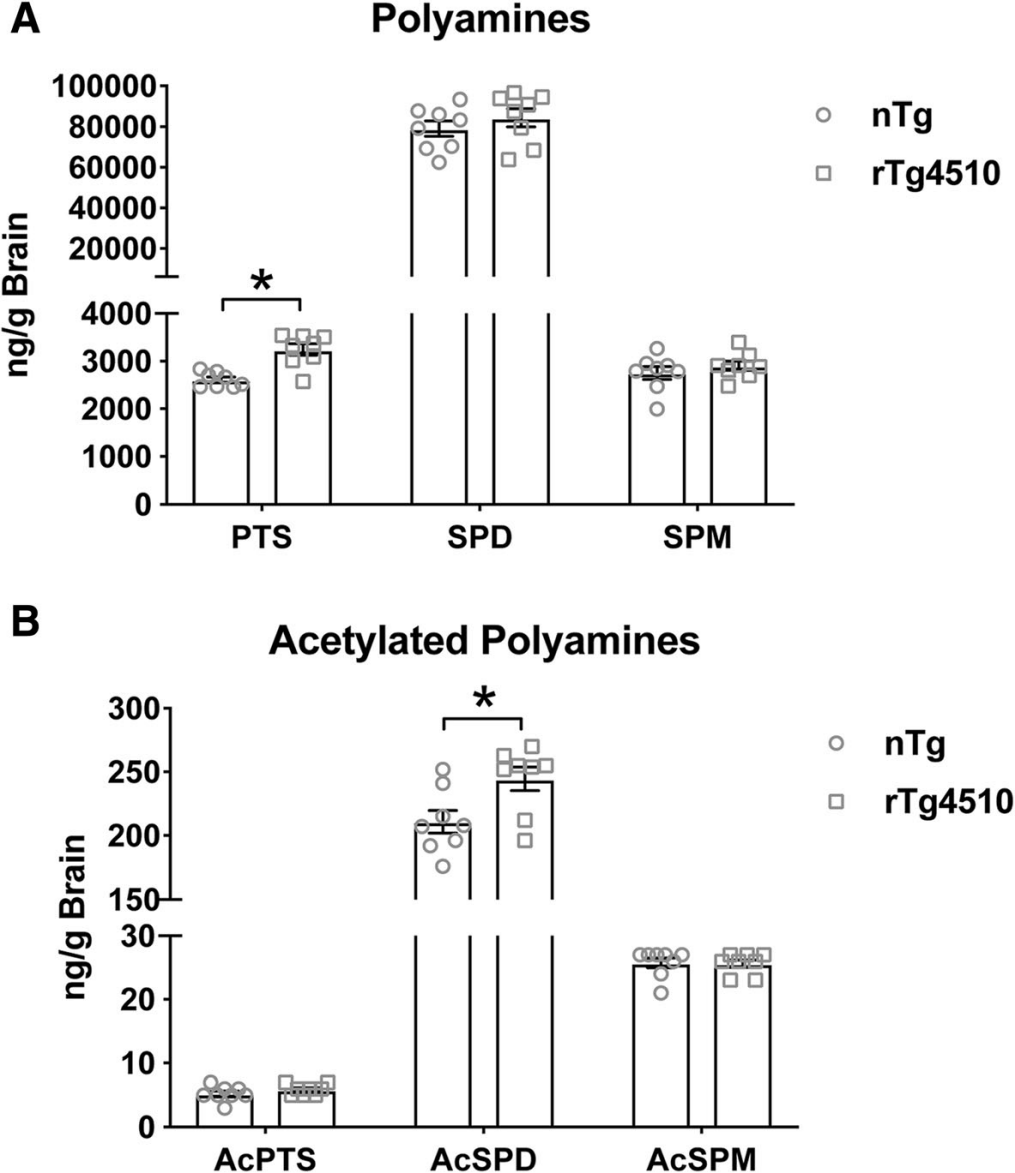
**a**, **b** Polyamine (putrescine (PTS: *t*(13) = − 7.687, *p* = .000), spermidine (SPD: *t* (13) = − .662, *p* = .520), spermine (SPM: *t*(13) = − 1.133, *p* = .278)) and acetylated polyamine (acetylputrescine (AcPTS: *t*(13) = 1.848, *p* = .087), acetylspermidine (AcSPD: *t*(13) = − 3.238, *p* = .006), acetylspermine (AcSPM: *t*(13) = − .052, *p* = .959)) quantification of brain homogenates from 8-month-old nTg and rTg4510 mice (*n* = 8). Independent samples *t* test *p < .05. Data is represented by means ± S.E.M.

To determine the extent of end-product polyamine levels, we measured polyamines and their acetylated products in rTg4510 mice (Fig. 2a, b). Although many of the polyamine enzyme levels were altered in some way, only putrescine and acetylspermidine were significantly increased in the brains of rTg4510 mice (Fig. 2a, b), compared to non-transgenic littermates. An increase in both products indicates evidence of the SSAT-induced polyamine back conversion revealing a signature of the tau-PSR. Notably, increased putrescine in rTg4510 does not account from increased metabolism of ornithine (the precursor substrate for putrescine), because ODC decreased in tau transgenic mice. This suggests sustained SSAT-dependent polyamine back conversion as one potential mechanism for the tau-PSR. To date, acetylation of spermidine (and other polyamines) has only been shown to occur under the control of SSAT, signifying the impact of SSAT dysregulation.

### Polyamines and acetylated polyamines differentially impact tau fibrillization

**Fig. 3:**
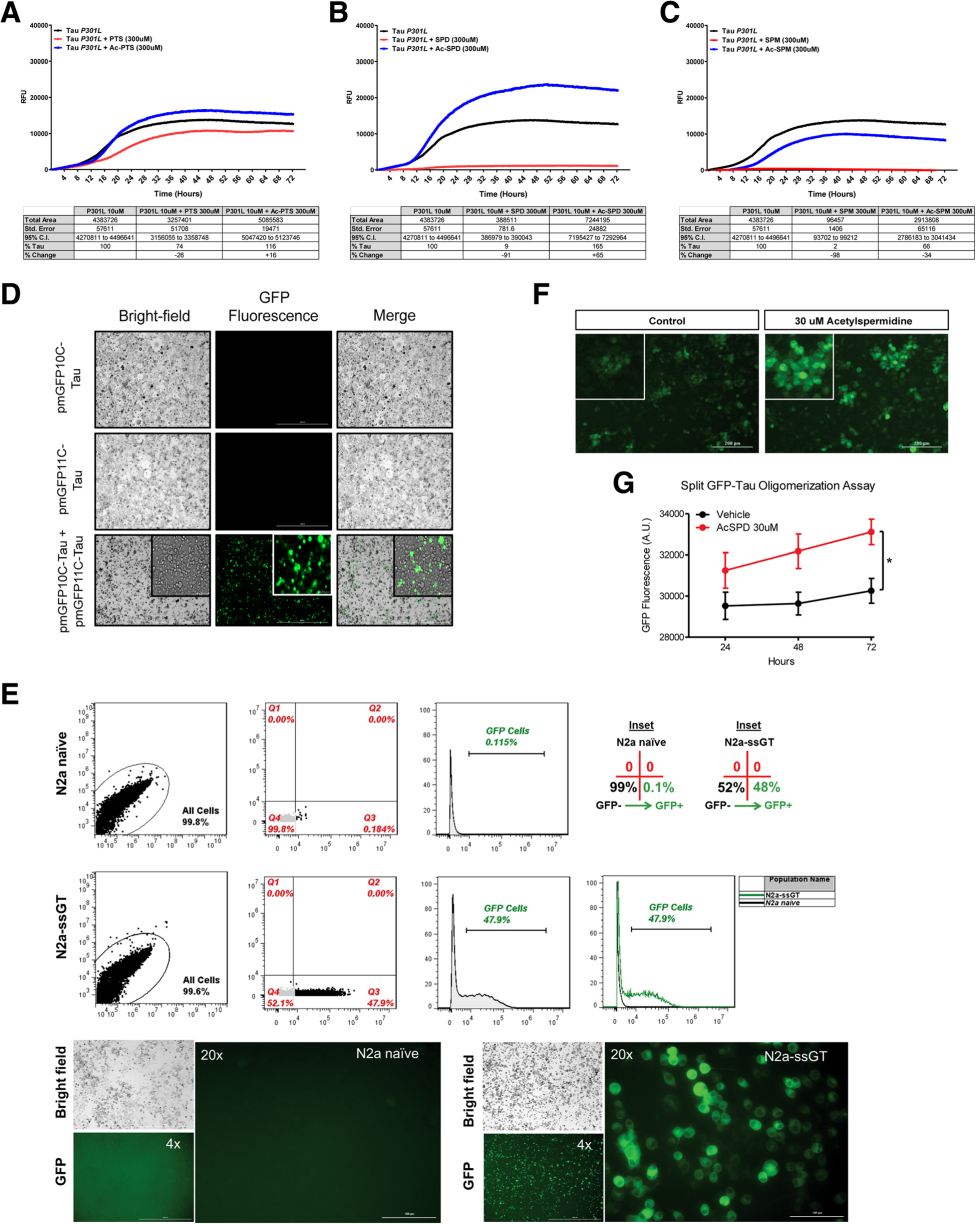
Polyamines and acetylated polyamines differentially impact tau fibrillization and acetylspermidine increases tau oligomerization. **a**–**c** Thioflavin T assay using recombinant 4R0N (P301L) tau and treatment of polyamines (putrescine, spermidine, spermine) or acetylated polyamines (acetylputrescine, acetylspermidine, acetylspermine) followed by quantification of area under the curve (AUC) compared to tau + vehicle controls. Data is represented as triplicate average and tables reflect AUC change from tau + vehicle control (% tau, % change). **d**, **e** Characterization and validation of split GFP-Tau individual plasmids (pmGFP10C-Tau, pmGFP11C-Tau) and monoclonal cell line (N2a-ssGT). **f**, **g** Representative images (at 24 h) and quantification of tau oligomerization following treatment with acetylspermidine. Simple main effects analysis showed that 30 μM acetylspermidine increased tau oligomerization (*F*(1, 78) = 7.140, *p* = .009. No effect of time (*F*(2,78) = .719, *p* = .490) or interaction of variables (*F*(2, 78) = .144, *p* = .866) was detected. 2 × 3 Factorial analysis of variance (ANOVA), followed by post hoc comparisons using Fishers PLSD. *p < .05, the asterisk inidcates the main effect of drug. Data is represented by means ± S.E.M.

To determine whether polyamines and acetylated polyamines directly impact tau fibrillization, we co-incubated recombinant 4R0N (P301L) tau with polyamines and acetylated polyamines (300 μM) or vehicle control in solution using the ThT assay (Fig. 3a–c). Polyamines prevented tau fibrillization, as measured by area under the curve (AUC), with higher-order polyamines (longer chain length) inhibiting fibrillization to greater extent and with greater potency. Strikingly, both spermidine and spermine appear capable of completely blocking tau fibrillization in this system. Interestingly, acetylated polyamines displayed a different profile for tau fibrillization. Specifically, acetylspermidine failed to inhibit fibrillization of tau and actually increased fibrillization. In addition, acetylputrescine and acetylspermine failed to reduce tau fibrillization to the same extent as putrescine and spermine respectively, suggesting different impacts of polyamines versus acetylated polyamines on tau biology.

### Acetylspermidine increases tau oligomerization

To further determine whether increases in acetylspermidine could be contributing to disease progression, we developed a monoclonal superfolder split GFP-tau cell line (murine neuroblastoma; N2a-ssGT) to measure tau oligomerization. Characterization and validation of individual plasmids were performed. Subsequently, characterization and validation of stably expressed tau in a monoclonal N2a-ssGT cell line was conducted by measurement of GFP fluorescence by Cytation™ 3 Cell Imaging Multi-Mode Reader (BioTek™) and Accuri® C6 flow cytometer (BD Biosciences) (Fig. 3d, e, respectively). N2a-ssGT cells were incubated with acetylspermidine for 24, 48, and 72 h, and tau oligomerization was measured by GFP fluorescence. In this model, the SSAT-dependent byproduct acetylspermidine increased tau oligomerization (Fig. 3f, g). Although it is difficult to predict the various endogenous concentrations of polyamines and acetylated polyamines within subcellular compartments and the impact on tau biology, these data signify a detrimental role for accumulation of acetylated products, namely acetylspermidine, on tau fate.

### AAV9 Tau ΔD421 overexpression induces a tau phenotype and tau-PSR: SSAT disruption prevents the tau-PSR and partially reduces phospho-tau but produces an independent behavioral phenotype

To determine the impact of SSAT disruption on the tau-PSR, we overexpressed tau ΔD421 (AAV9 Tau ΔD421) in SSAT null mice (SSAT-/-). This post-translational modification was chosen based on its observed expression in rTg4510 mice (Fig. 1d–h) as well as its ability to enhance the spreading of tau pathology [61], assemble more rapidly and more extensively into tau filaments than wild-type tau in vitro [10], and produce cognitive impairment [62].

**Fig. 4:**
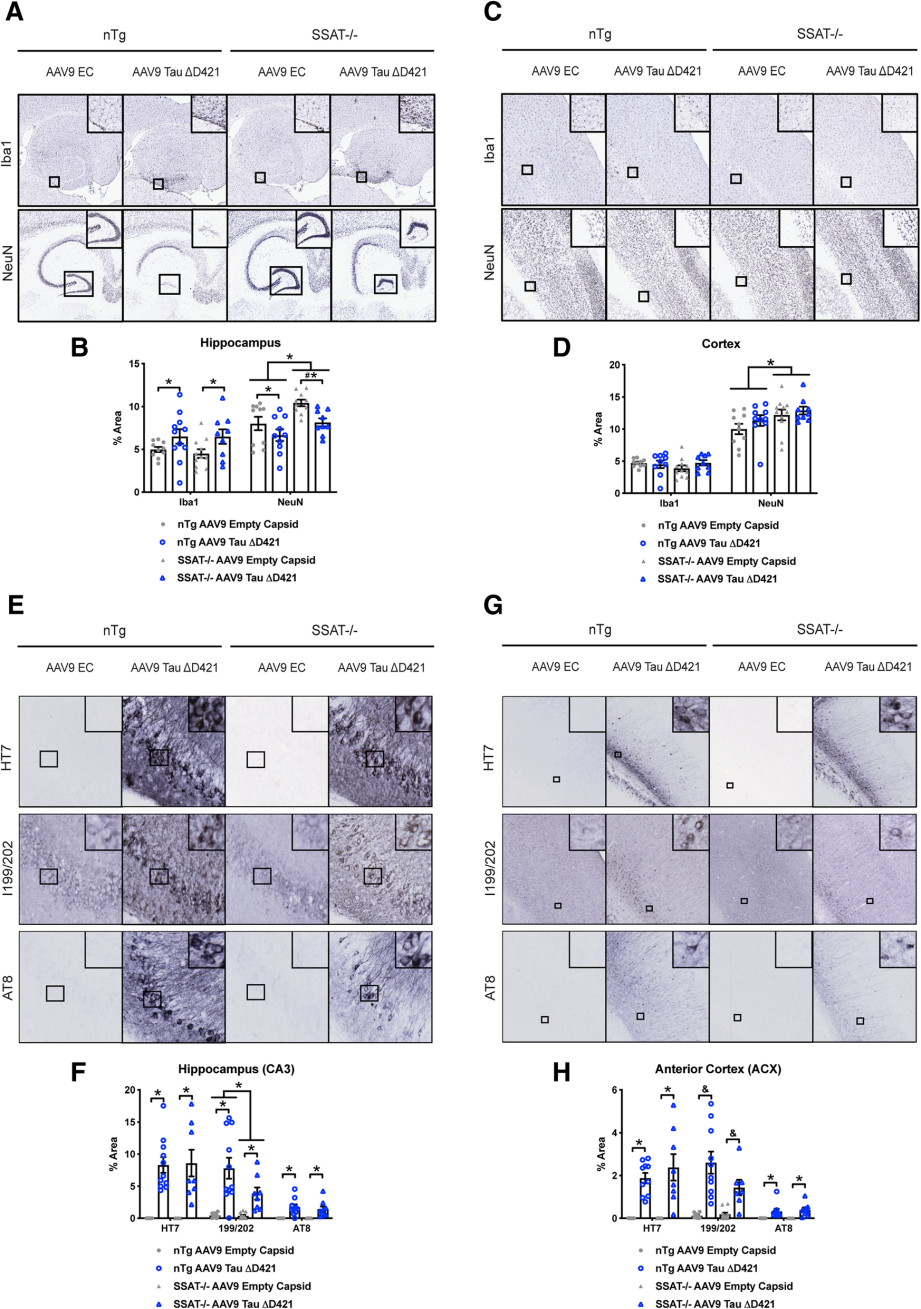
AAV9 Tau ΔD421 produces hippocampal inflammation and targeted disruption of SSAT increases NeuN expression and reduces tau neuropathology. **a**–**d** Representative images and quantification of immunohistochemical analysis of hippocampal and cortical inflammation (Iba1) and NeuN expression in response to 4-month incubation of either AAV9 empty capsid (EC) or AAV9 Tau ΔD421 in 15-month-old nTg and SSAT-/- mice (*n* = 7–11). Iba1: Simple main effects analysis showed that AAV9 Tau ΔD421 significantly increased inflammation (Iba1) in only the hippocampus (*F*(1, 36) = 6.823, *p* = .013). Further, within each genotype, pairwise comparisons revealed a significant difference in hippocampal Iba1 between only the SSAT-/- AAV9 empty capsid and SSAT-/- AAV9 Tau ΔD421 groups (*p* = .048). NeuN: Simple main effects analysis showed that AAV9 Tau ΔD421 significantly decreased NeuN expression (NeuN) in only the hippocampus (*F*(1, 35) = 8.973, *p* = .005). Interestingly, simple main effects analysis also showed that SSAT-/- mice had significantly increased NeuN expression (NeuN) in both the hippocampus (*F*(1, 39) = 10.343, *p* = .003) and the cortex (*F*(1, 35) = 8.100, *p* = .007), relative to non-transgenic litter-mates. Further, within each genotype, pairwise comparisons revealed a significant difference in hippocampal NeuN between only the SSAT-/- AAV9 empty capsid and SSAT-/- AAV9 Tau ΔD421 groups (*p* = .013). **e**–**h** Representative images and quantification of immunohistochemical analysis of hippocampal (CA3) and anterior cortex (ACX) tau neuropathology in response to 4-month incubation of either AAV9 empty capsid (EC) or AAV9 Tau ΔD421 in 15-month old nTg and SSAT-/- mice (*n* = 7–11). HT7: Simple main effect analysis showed that AAV9 Tau ΔD421 significantly increased total tau (HT7) in both the CA3 of the hippocampus (*F*(1, 34) = 55.939, *p* = .000) and the anterior cortex (ACX; *F*(1, 35) = 57.020, *p* = .000). Importantly, the level of total tau was not significantly different between nTg and SSAT-/- mice, ensuring treatment was equal across groups. pSer199/202: Simple main effects analysis showed that AAV9 Tau ΔD421 significantly increased Tau pSer199/202 in the CA3 (*F*(1, 36) = 29.869, *p* = .000) and the ACX (*F*(1, 34) = 36.192, *p* = .000). Interestingly, in the CA3, there was also a main effect of genotype (*F*(1, 36) = 4.114, *p* = .05), and pairwise comparison revealed a significant difference between nTg AAV9 Tau ΔD421 and SSAT-/- AAV9 Tau ΔD421 mice (*p* = .010), identifying a protective effect of SSAT disruption in CA3 Tau pSer199/202. Further, in the ACX, there was a significant interaction of factors (*F*(1, 34) = 3.994, *p* = .05), and pairwise comparison again revealed a significant difference between nTg AAV9 Tau ΔD421 and SSAT-/- AAV9 Tau ΔD421 mice (*p* = 0.17), identifying a protective effect of SSAT disruption in ACX Tau pSer 199/202. AT8: Simple main effects analysis showed that AAV9 Tau ΔD421 significantly increased phosphorylated paired helical filament tau (PHF; AT8) in both the CA3 (*F*(1, 36) = 32.677, *p* = .000) and ACX (*F*(1, 33) = 22.792, *p* = .000). No effect of genotype or interaction of factors was detected. 2 × 2 Factorial analysis of variance (ANOVA), followed by pairwise comparisons using Fisher’s PLSD. *p < .05; asterisks indicate the main effect of treatment and main effect of genotype, and ampersands indicate the interaction of genotype and treatment. Data is represented by means ± S.E.M.

Immunohistochemical analyses revealed AAV9 Tau ΔD421 increased hippocampal, but not cortical, microglial activation (Iba1); however, SSAT disruption did not impact any of these measurements (Fig. 4a–d). Interestingly, AAV9 Tau ΔD421 decreased hippocampal NeuN expression in both genotypes compared to AAV9 empty capsid; however, SSAT disruption significantly increased hippocampal and cortical NeuN expression (Fig. 4a–d).

Relative to injection sites, AAV9 Tau ΔD421 increased total tau (HT7) in the CA3 of the hippocampus and anterior cortex (Fig. 4e–h) and importantly was not significantly different between nTg and SSAT-/- mice, ensuring equal tau expression across genotypes. Examining phospho-tau epitopes within these regions, analyses revealed that targeted SSAT disruption reduced both hippocampal and cortical tau pSer199/202, but not PHF tau (AT8) (Fig. 4e–h), indicating a discrete reduction in tau phospho epitopes by targeting the tau-PSR through SSAT.

**Fig. 5:**
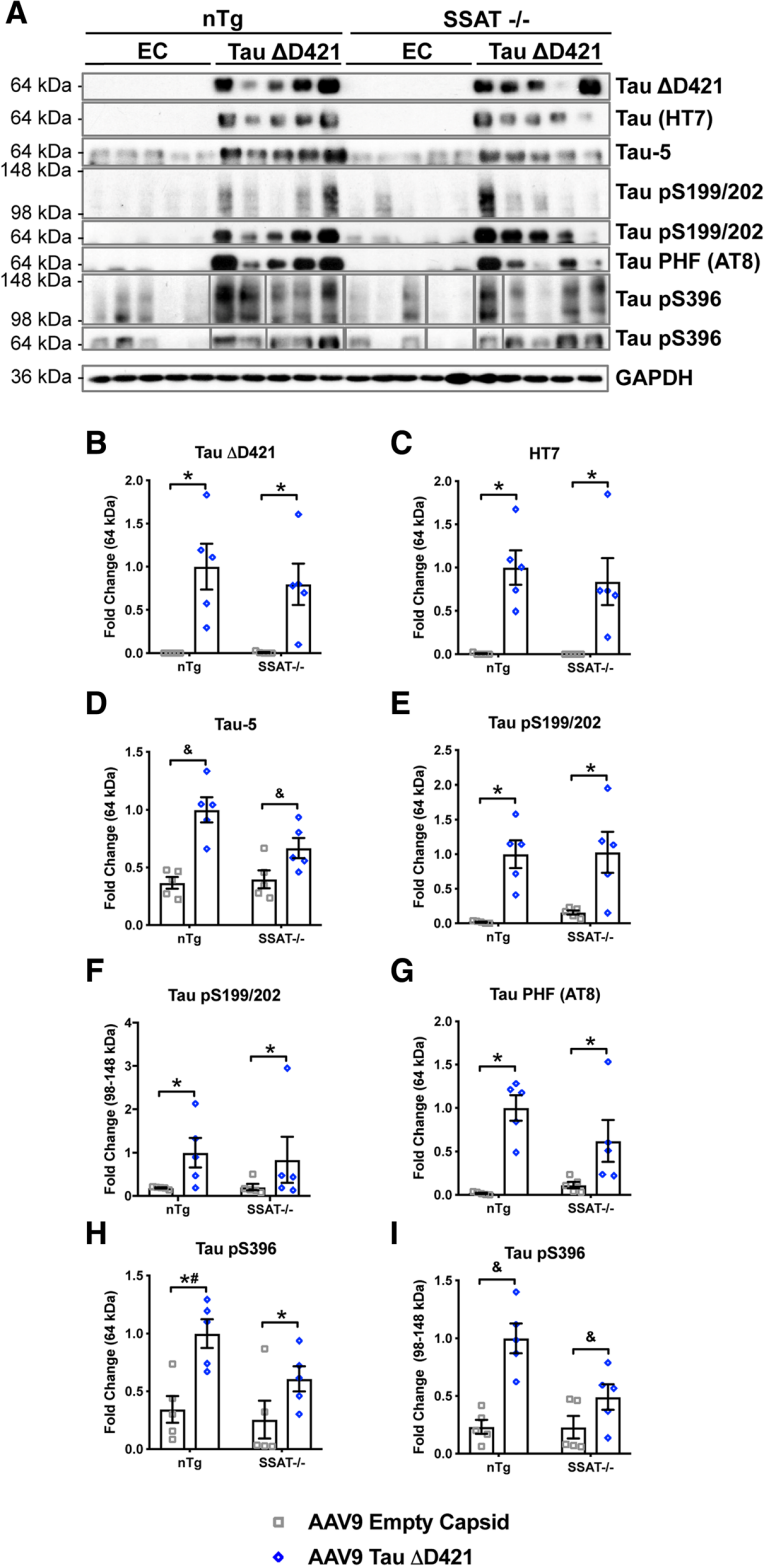
AAV9 Tau ΔD421 produces robust hippocampal tau neuropathology; SSAT disruption prevents the accumulation of high molecular weight tau phospho-epitopes. **a**–**i** Representative images and quantification of western blot analysis of hippocampal tau neuropathology, exogenous Tau ΔD421, exogenous total tau (HT7), exogenous and endogenous total tau (Tau-5), pS199/202, tau-paired helical filament (Tau PHF; AT8), and pS396, in response to 4-month incubation of either AAV9 empty capsid (EC) or AAV9 Tau ΔD421 in 15-month-old nTg and SSAT-/- mice (*n* = 3–5). Tau ΔD421: Simple main effects analysis showed that AAV9 Tau ΔD421 significantly increased Tau ΔD421 (*F*(1, 16) = 24.925, *p* = .000). Importantly, the level of Tau ΔD421 was not significantly different between nTg and SSAT-/- mice, ensuring treatment was equal across groups. No main effect of genotype or interaction of factors was detected on Tau ΔD421. Total tau (HT7): Simple main effects analysis showed that AAV9 Tau ΔD421 significantly increased total tau (HT7) (*F*(1, 16) = 29.585, *p* = .000). Importantly, the level of total tau was not significantly different between nTg and SSAT-/- mice, ensuring treatment was equal across groups. No main effect of genotype or interaction of factors was detected on total tau (HT7). Tau-5: An interaction of factors was detected (*F*(1, 16) = 4.657, *p* = .046) on Tau-5, indicating the levels of Tau-5 are dependent on genotype, with targeted SSAT disruption decreasing Tau-5 levels. Tau pS199/202: Simple main effects analysis showed that AAV9 Tau ΔD421 significantly increased monomeric (*F*(1, 16) = 26.621, *p* = .000) and high molecular weight (HMW; *F*(1, 16) = 5.143, *p* = .038) Tau pS199/202. No main effect of genotype or interaction of factors was detected on pS199/202. AT8: Simple main effects analysis showed that AAV9 Tau ΔD421 significantly increased Tau PHF (AT8; *F*(1, 16) = 27.482, *p* = .000). No main effect of genotype or interaction of factors was detected on AT8. Tau pS396: Simple main effects analysis showed that AAV9 Tau ΔD421 significantly increased monomeric (*F*(1, 16) = 15.084, *p* = .001) Tau pS396; however, pairwise comparisons revealed that AAV9 Tau ΔD421 only significantly increased monomeric pS396 in the nTg genotype (*p* = .003) and not in the SSAT-/- genotype (*p* = .073) indicating an impact of SSAT on monomeric Tau pS396. This notion is supported by the significant pairwise comparison between nTg AAV9 Tau ΔD421 and SSAT-/-0 AAV9 Tau ΔD421 groups (*p* = .048). Further, there was an interaction of factors on HMW Tau pS396 (*F*(1, 16) = 25.183, *p* = .000), indicating the levels of HMW Tau pS396 are dependent on genotype, with targeted SSAT disruption decreasing HMW pS396 levels. This is supported by the lack of significant pairwise comparison between SSAT-/- AAV9 empty capsid and SSAT-/- AAV9 Tau ΔD421 (*p* = .09), and the significant pairwise comparison between nTg AAV9 Tau ΔD421 and SSAT-/- AAV9 Tau ΔD421, again with SSAT-/- decreasing HMW pS396 levels. 2 × 2 Factorial analysis of variance (ANOVA), followed by pairwise comparisons using Fishers PLSD. *p < .05; asterisks indicate the main effect of treatment and main effect of genotype, ampersands indicate the interaction of genotype and treatment, and number signs indicate the pairwise comparison within genotype. Data is represented by means ± S.E.M.

Western blot analyses confirmed the level of tau ΔD421 (Fig. 5a, b) and total tau (HT7; Fig. 5a, c) were not significantly different between nTg and SSAT-/- mice, confirming that human tau expression was equal across genotypes. Further, analyses revealed SSAT disruption reduced the aggregation of monomeric and high-molecular weight phospho-tau ser396 (tau pSer396) (Fig. 5a, h, i), suggesting that SSAT disruption discretely impacts the level of phospho-tau species and certain forms of tau associated with the Tau-5 epitope (Fig. 5a, d, h, i).

**Fig. 6:**
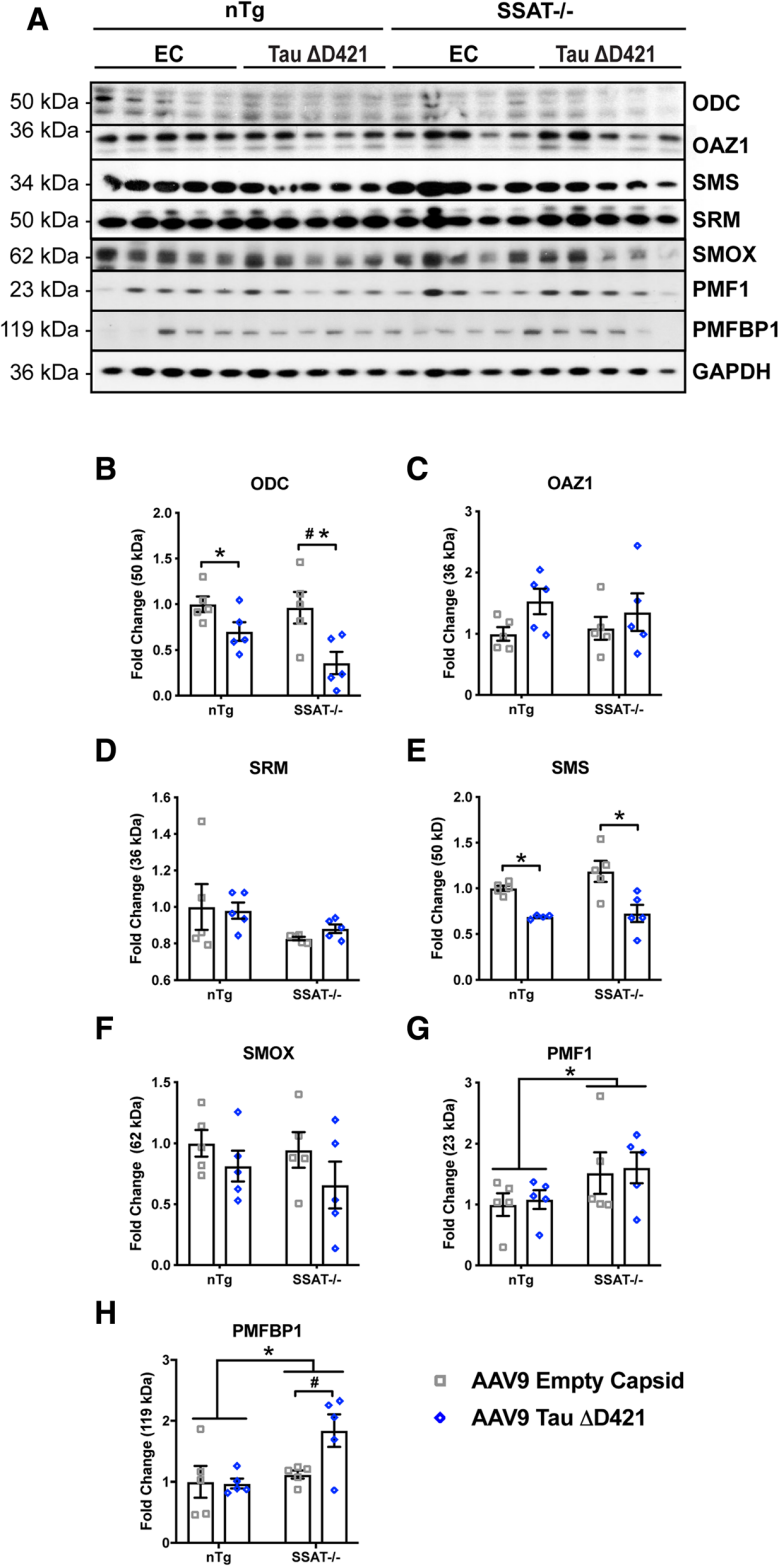
AAV9 AAV9 Tau ΔD421 induces tau-PSR; SSAT disruption prevents polyamine dysregulation. **a**–**h** Representative images and quantification of western blot analysis of hippocampal polyamine dysregulation (ODC, OAZ1, SRM, SMS, SMOX, PMF1, and PMFBP1) in response to 4-month incubation of either AAV9 empty capsid (EC) or AAV9 Tau ΔD421 in 15-month-old nTg and SSAT-/- mice (*n* = 4–5). ODC: Simple main effects analysis showed that AAV9 Tau ΔD421 significantly decreased ornithine decarboxylase (ODC) (*F*(1, 16) = 13.164, *p* = .002). Further, within each genotype, pairwise comparisons revealed a significant reduction of ODC by AAV9 Tau ΔD421 in SSAT-/- mice (*p* = .003); an effect that was absent in non-transgenic mice. No main effect of genotype (*F*(1, 16) = 2.358, *p* = .144) or interaction of factors (*F*(1, 16) = 1.511, *p* = .237) was detected on ODC. OAZ1: No main effect of genotype (*F*(1, 16) = .040, *p* = .844), AAV9 Tau ΔD421 (*F*(1, 16) = 3.430, *p* = .083), or interaction of factors (*F*(1, 16) = .388, *p* = .542) was detected in OAZ1. SRM: No main effect of genotype (*F*(1, 16) = 4.035, *p* = .062), AAV9 Tau ΔD421 (*F*(1, 16) = .064, *p* = .804), or interaction of factors (*F*(1, 16) = .300, *p* = .592) was detected in SRM. SMS: Simple main effects analysis showed that AAV9 Tau ΔD421 significantly decreased spermine synthase (SMS) (*F*(1, 15) = 23.066, *p* = .000). No effect of genotype (*F*(1, 15) = 1.918, *p* = .186) or interaction of factors (*F*(1, 15) = .850, *p* = .371) was detected on SMS. SMOX: No main effect of genotype (*F*(1, 16) = .519, *p* = .482), AAV9 Tau ΔD421 (*F*(1, 16) = 2.604, *p* = .126), or interaction of factors (*F*(1, 16) = .121, *p* = .733) was detected in SMOX. PMF1: Simple main effects analysis showed that SSAT disruption significantly increased polyamine-modulated factor 1 (PMF1) (*F*(1, 16) = 4.522, *p* = .049). No main effect of AAV9 Tau ΔD421 (*F*(1, 16) = .114, *p* = .740) or interaction of factors (*F*(1, 16) = .000, *p* = .993) was detected in PMF1. PMFBP1: Simple main effects analysis showed that SSAT disruption significantly increased polyamine-modulated factor binding protein 1 (PMFBP1) (*F*(1, 16) = 6.499, *p* = .021). Further, within each genotype, while there was no simple main effect of AAV9 Tau ΔD421 (*F* (1,16) = 3.248, *p* = .090), pairwise comparison revealed a significant difference in PMFBP1 between only the SSAT-/- AAV9 empty capsid and SSAT-/- AAV9 Tau ΔD421 groups (*p* = .017), identifying a SSAT-dependent effect of AAV9 Tau ΔD421 on PMFBP1. There was no significant interaction of factors (*F*(1, 16) = 3.786, *p* = .069) on PMFBP1. 2 × 2 Factorial analysis of variance (ANOVA), followed by pairwise comparisons using Fishers PLSD. *p < .05; asterisks indicate the main effect of treatment and main effect of genotype, number signs indicate the pairwise comparison within genotype. Data is represented by means ± S.E.M.

Furthermore, AAV9 Tau ΔD421 produced a unique tau-PSR (Fig. 6a–h). Similar to that of rTg4510 mice, AAV9 Tau ΔD421 decreased polyamine anabolic enzymes ODC and SMS (Fig. 6a, e). One enzyme capable of binding ODC to render its degradation by the proteasome is ornithine decarboxylase antizyme1 (OAZ1 or AZ). Although the mean average was higher in AAV9 Tau ΔD421 mice, OAZ1 failed to reach statistical significance (Fig. 6c). Additionally, SRM and SMOX show no change between groups (Fig. 6d, f), suggesting that tau induces a response specific to ODC and SMS. Interestingly, PMF1 significantly increased in SSAT-/- mice (Fig. 6g), possibly as a compensatory mechanism for SSAT deficiency, whereas PMFPBP1 was only significantly induced in SSAT-/- mice that received AAV9 Tau ΔD421 (Fig. 6h) suggesting a unique interaction of tau pathology and SSAT regulation. These interactions between the tau phenotype and enzymatic signatures warrant further investigation.

**Fig. 7:**
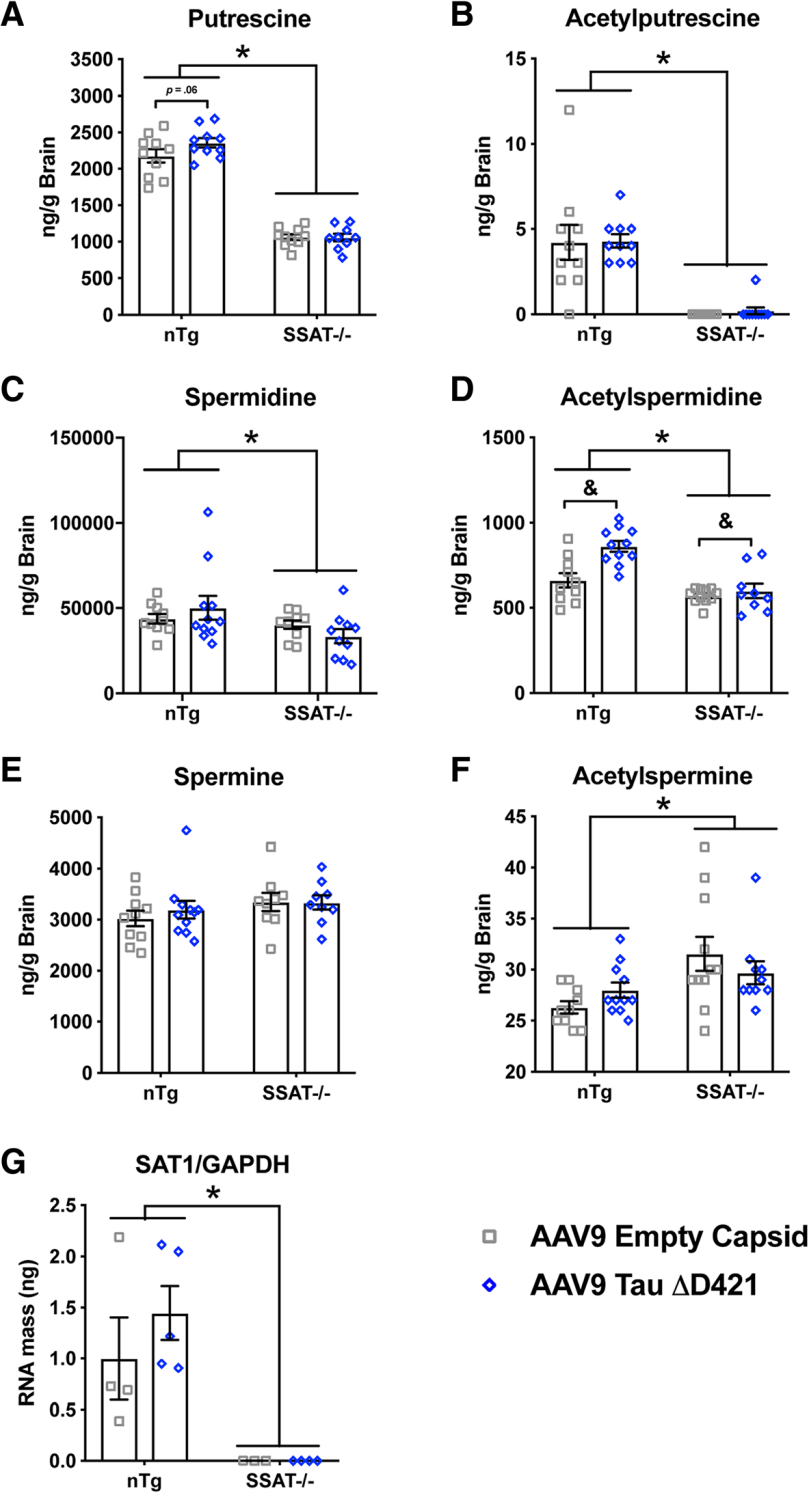
Polyamine quantification (putrescine, spermidine, acetylputrescine, acetylspermidine) of the brain homogenates in response to 4-month incubation of either AAV9 empty capsid (EC) or AAV9 Tau ΔD421 in 15-month-old nTg and SSAT-/- mice (*n* = 9–11). **a** Putrescine: Simple main effects analysis showed that SSAT disruption significantly reduced putrescine (*F*(1, 35) = 332.618, *p* = .000). Further, within each genotype, pairwise comparison revealed a trend in difference in putrescine between only the nTg AAV9 empty capsid and nTg AAV9 Tau ΔD421 groups (*p* = .061). No main effect of AAV9 Tau ΔD421 (*F*(1, 35) = 1.698, *p* = .201) or interaction of factors (*F*(1, 35) = 1.960, *p* = .170) was detected on putrescine. **b** Acetylputrescine: Simple main effects analysis showed that SSAT disruption significantly reduced acetylputrescine (*F*(1, 37) = 57.551, *p* = .000). No main effect of AAV9 Tau ΔD421 (*F*(1, 37) = .075, *p* = .785) or interaction of factors (*F*(1, 37) = .008, *p* = .928) was detected on acetylputrescine. **c** Spermidine: Simple main effects analysis showed that SSAT disruption significantly reduced spermidine (*F*(1, 37) = 4.736, *p* = .036). No main effect of AAV9 Tau ΔD421 (*F*(1, 37) = .001, *p* = .970) or interaction of factors (*F*(1, 37) = 2.009, *p* = .165) was detected on spermidine. **d** Acetylspermidine: Simple main effects of genotype (*F*(1, 37) = 27.723, *p* = .000), AAV9 Tau ΔD421 (*F*(1, 37) = 11.661, *p* = .002) and interaction of factors (*F*(1, 37) = 6.539, *p* = .015) was detected on acetylspermidine. **e** Spermine: No main effect of genotype (*F*(1, 35) = 2.012, *p* = .165), AAV9 Tau ΔD421 (*F*(1, 35) = .236, *p* = .630), or interaction of factors (*F*(1, 35) = .296, *p* = .590) was detected on Spermine. **f** Acetylspermine: Simple main effects analysis showed that SSAT disruption significantly increased acetylspermine (*F*(1, 38) = 9.360, *p* = .004). No main effect of AAV9 Tau ΔD421 (*F*(1, 38) = .004, *p* = .949) or interaction of factors (*F*(1, 38) = 2.439, *p* = .127) was detected on acetylspermine. **g** SSAT mRNA: mRNA expression of SSAT in response to 4-month incubation of either AAV9 empty capsid (EC) or AAV9 Tau ΔD421 in 15-month old nTg and SSAT-/- mice (*n* = 3–5). Simple main effects showed that SSAT disruption significantly reduced SSAT expression, normalized to GAPDH, (*F*(1, 12) = 20.660, *p* = .001. No effect of AAV9 Tau ΔD421 (*F*(1, 12) = .687, *p* = .423) or interaction of factors (*F*(1, 12) = .694, *p* = .421) was detected on SSAT expression. 2 × 2 Factorial analysis of variance (ANOVA), followed by pairwise comparisons using Fishers PLSD. *p < .05; asterisks indicate the main effect of treatment and main effect of genotype, ampersands indicate the interaction of genotype and treatment, and number signs indicate the pairwise comparison within genotype. Data is represented by means ± S.E.M.

Regarding polyamines and their metabolites, SSAT-/- mice in general displayed reduced putrescine, acetylputrescine, spermidine, and acetylspermidine (Fig. 7a–d; Additional file 1: Figure S1 a-g). Importantly, AAV9 Tau ΔD421 increased acetylspermidine and showed a trend toward increased putrescine whereas SSAT deficiency prevented these effects (Fig. 7a, d). Conversely, spermine showed no change between groups whereas acetylspermine was slightly but still significantly elevated (Fig. 7e–f). These data suggest that SSAT disruption does not completely eliminate all acetylated products but primarily impacts polyamine retro-conversion, recycling, and homeostasis of polyamine levels. It also suggests that unknown and alternative acetylation processes may exist, specifically in regard to spermine. Furthermore, tau seemingly promotes SSAT-induced back-conversion that can be measured at the level of acetylated polyamines, namely acetylspermidine (Fig. 7d). Of note, spermidine shows the largest abundance of all three polyamines in the brain and likely outcompetes for SSAT as a primary substrate; therefore, acetylated spermidine increases in response to SSAT induction elicited by tau. Curiously, while qRT-PCR confirmed SAT1 disruption in SSAT-/- mice, AAV9 Tau ΔD421 did not significantly increase SAT1 expression (Fig. 7g), as the elevations in back-converted putrescine and acetylspermidine would predict (Fig. 7a, d).

**Fig. 8:**
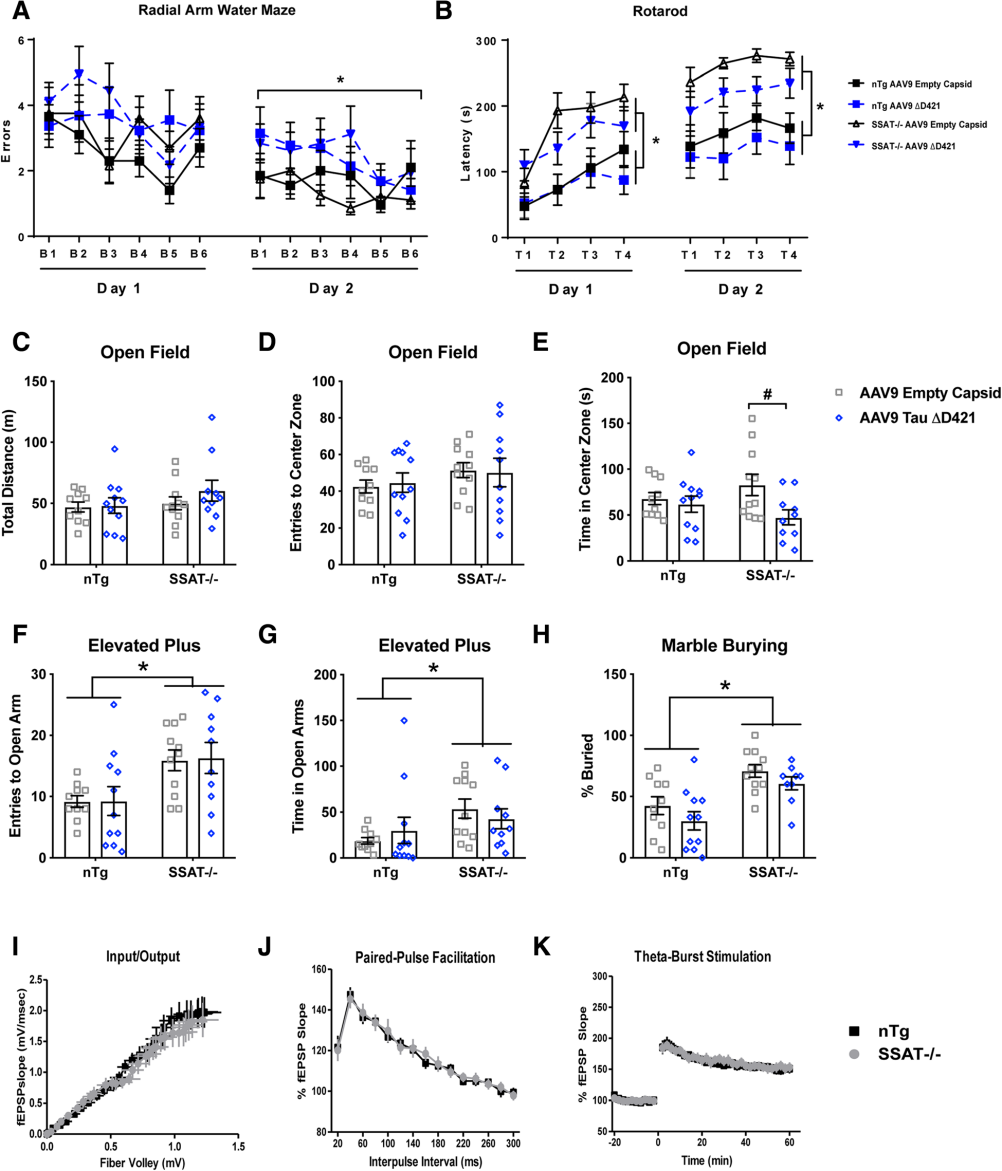
AAV9 Tau ΔD421 produces cognitive impairment; SSAT disruption produces behavioral phenotype. **a** Radial arm water maze errors for per block and per day of 15-month-old nTg and SSAT-/- mice administered either AAV9 empty capsid (EC) or AAV9 Tau ΔD421 (*n* = 9–11). Simple main effects analysis showed that AAV9 Tau ΔD421 significantly increased errors (*F*(1, 36) = 5.204, *p* = .029) on day 2 of the radial arm water maze (RAWM). No effect of genotype (*F*(1, 36) = 1.521, *p* = .225), AAV9 Tau ΔD421 (*F*(1, 36) = 3.196, *p* = .082), or interaction of factors (*F*(1, 36) = .392, *p* = .535) was found on errors on day 1 of RAWM. No effect of genotype (*F*(1, 36) = .051, *p* = .822) or interaction of factors (*F*(1, 36) = .525, *p* = .473) was found on errors on day 2 of RAWM. Repeated measures mixed analysis of variance (ANOVA), *p < .05. Data is represented by means ± S.E.M. Asterisks indicate the main effect of treatment. **b** Rotarod performance latency per trial and per day of 15-month-old nTg and SSAT-/- mice administered either AAV9 empty capsid (EC) or AAV9 Tau ΔD421 (*n* = 9–11). Simple main effects analysis showed that SSAT disruption significantly increased latency to fall on day 1 (*F*(1, 37) = 14.164, *p* = .001) and day 2 (*F*(1, 37) = 19.626, *p* = .000) of the rotarod task. No effect of AAV9 Tau ΔD421 on day 1 or day 2 (*F*(1, 37) = .779, *p* = .383; *F*(1, 37) = 2.985, *p* = .092), respectively), or interaction of factors on day 1 or day 2 (*F*(1, 37) = .080, *p* = .779; *F*(1, 37) = .149, *p* = .702, respectively) was detected on rotarod latency to fall. Repeated measures mixed analysis of variance (ANOVA), *p < .05. Data is represented by means ± S.E.M. Asterisks indicate the main effect of genotype. **c**–**h** Behavioral assessment in the open field, elevated plus maze, and marble burying tasks of 15-month-old nTg and SSAT-/- mice administered either AAV9 empty capsid (EC) or AAV9 Tau ΔD421 (*n* = 9–11). 2 × 2 Factorial analysis of variance (ANOVA), followed by pairwise comparisons using Fishers PLSD. *p < .05; Asterisks indicate the main effect of genotype and number signs indicate the pairwise comparison within genotype. Data is represented by means ± S.E.M. **c**–**e** Open field: Simple main effects analysis showed no effect of SSAT disruption on total distance traveled (*F*(1, 38) = 1.488, *p* = .230), number of entries to the center zone (*F*(1, 38) = 1.825, *p* = .185), or time in the center zone (*F*(1, 38) = .001, *p* = .970). While there was no effect of AAV9 Tau ΔD421 on total distance traveled (*F*(1, 38) = .844, *p* = .364) or number of entries to the center zone (*F*(1, 38) = .007, *p* = .935), there was a main effect of treatment on time in the center zone in that AAV9 Tau ΔD421 significantly decreased time in the center zone (*F*(1, 38) = 5.065, *p* = .030); however, pairwise comparisons revealed this effect was only present in SSAT-/- mice (*p* = .01), suggesting a modest interaction of genotype and treatment on anxiety-like behavior as measured by this task. **f**, **g** EPM: Simple main effects analysis showed that SSAT disruption significantly increased entries to the open arm (*F*(1, 38) = 11.894, *p* = .001) and time in the open arm (*F*(1, 38) = 4.892, *p* = .033). No main effect of AAV9 Tau ΔD421 (*F*(1, 3) = .014, *p* = .908; *F*(1, 38) = .001, *p* = .982, respectively) or interaction of factors (*F*(1, 38) = .006, *p* = .937; *F*(1, 38) = 1.070, *p* = .307, respectively) was detected on EPM open arm entries and open arm time. **h** Marble burying: Simple main effects analysis showed that SSAT disruption significantly increased percent of marbles buried (*F*(1, 37) = 20.576, *p* = .000). No main effect of AAV9 Tau ΔD421 (*F*(1, 37) = 3.034, *p* = .090) or interaction of factors (*F*(1, 37) = .029, *p* = .866) was detected on percent marbles buried. **i**–**k** Comparing nTg and SSAT-/- mice (*n* = 6), normal input/output curve at Schaffer collateral/CA1 synapses was observed. Nonlinear regression, line fits were compared using the extra sum-of-squares *F* test for the Fiber volley (*p* = 0.4907) and fEPSP (*p* = 0.1019), respectively. No difference was seen in short-term presynaptic plasticity measured by paired-pulse facilitation (PPF), (*F*(1, 48) = 0.010, *p* = 0.920.) LTP was induced in nTg and SSAT-/- slices using a theta-burst stimulation protocol and no significant differences were seen in the induction or maintenance of LTP in SSAT-/- compared to nTg slices (*F*(1, 12) = 0.642, *p* = 0.439)

AAV9 Tau ΔD421 also produced cognitive impairment in the radial arm water maze task (Fig. 8a); however, despite SSAT disruption’s ability to partially reduce specific phospho-tau epitopes (Fig. 4e–h, Fig. 5a, d, h, i) and prevent the tau-induced increases in putrescine and acetylspermidine (Fig. 7a, d), cognitive impairment induced by AAV9 Tau ΔD421 was not improved by SSAT disruption (Fig. 8a). Interestingly, a behavioral phenotype emerged as a product of SSAT disruption, characterized by increased hypervigilance in the rotarod task (Fig. 8b), decreased inhibition in the elevated plus maze (Fig. 8f, g), and increased compulsion in the marble burying task (Fig. 8h), compared to SSAT sufficient littermates, which may have influenced performance in the RAWM task. These data are highly intriguing given the role of SSAT in affective disorders [64–69]. Interestingly, AAV9 Tau ΔD421 significantly decreased time in the center zone of the open field task, but only in SSAT-/- mice (Fig. 8e), suggesting a modest interaction of SSAT disruption and AAV9 Tau ΔD421. To confirm that SSAT disruption alone did not influence learning and memory, we performed electrophysiology on hippocampal slices. The behavioral phenotype was not associated with any deficiencies in synaptic transmission or pre-synaptic short-term plasticity (Fig. 8i–k), solidifying behavioral changes in SSAT null mice as being rooted in affect rather than cognitive processing.

### siRNA SAT1 repression reduces tau aggregation

**Fig. 9:**
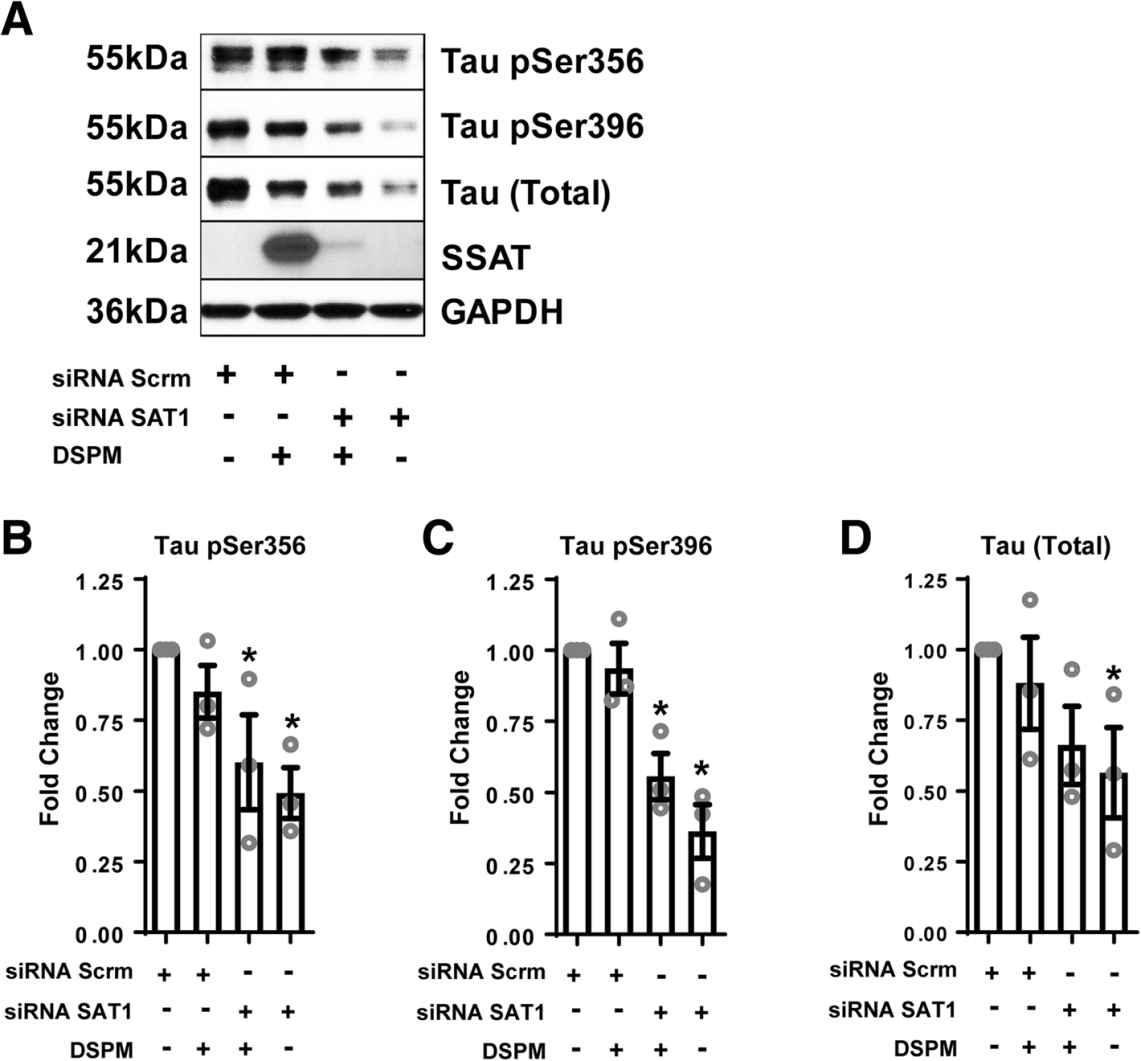
siRNA repression of SAT1 reduces tau pathology in vitro. **a** Representative images and quantification of western blot analysis of C3 HeLa cells stably overexpressing tau (4R0N) following transfection with siRNA scramble negative control or siRNA SAT1 (*n* = 3). **b** One way analysis of variance (ANOVA) revealed an effect of group on monomeric Tau pSer356 (*F*(3, 8) = 4.743, *p* = .035), and post hoc comparisons revealed a significant decrease in response to siRNA SAT1 with and without DSPM (*p* = .029, *p* = .010, respectively), compared to siRNA scrambled control. **c** One way analysis of variance (ANOVA) revealed an effect of group on monomeric Tau pSer396 (*F*(3, 8) = 15.829, *p* = .001), and post hoc comparisons revealed a significant decrease in response to siRNA SAT1 with and without DSPM (*p* = .003, *p* = .001, respectively), compared to siRNA scrambled control. **d** One way analysis of variance (ANOVA) revealed no effect of group on monomeric total tau (*F*(3, 8) = 2.236, *p* = .161); however, post hoc comparisons revealed a significant decrease in response to siRNA SAT1 without DSPM (*p* = .05), compared to siRNA scrambled control. One way analysis of variance (ANOVA), followed by post hoc comparisons using Fishers PLSD, *p < .05. Data is represented by means ± S.E.M.

Lastly, in an effort to better understand the impact of SSAT disruption on tau biology in vivo, we repressed SSAT using siRNA in C3 HeLa cells stably overexpressing tau (4R0N). Here, we show that SSAT repression also decreased monomeric total and phospho-tau levels compared to scrambled siRNA controls (Fig. 9a–d), further suggesting that simply modifying SSAT levels impacts tau biology.

## Discussion

Under normal conditions, tau stabilizes microtubules in neurons; however, in Alzheimer’s disease (AD) and tauopathies, tau becomes hyperphosphorylated, aggregates, and results in neuronal death. Tauopathies associate with inflammation [70–72], impaired synaptic function [73–75], and cognitive processing [3, 4]. Strategies for reducing disordered or aggregation-prone proteins found in AD, specifically tau, focus on targeting the tau phenotype. The tau phenotype includes, but is not limited to, the presence of tau species (phospho, high molecular weight oligomeric species, soluble, insoluble, neurofibrillary tangles, and other post translational modifications like truncation), inflammation, kinase activation, impaired autophagy, atrophy, neurodegeneration, and cognitive deficits [76, 77]. While numerous strategies attempt to reduce tau burden, there is no cure or treatments to slow or halt disease progression. In these studies, we measured a limited number of tau effects but were able to detect several changes in response to polyamine manipulation.

The ability of polyamines to improve learning and memory has been explored (see [33] for review); however, what remains to be elucidated is polyamine modulation (and their acetylated products) in response to chronic neuropathology. Despite being relatively unexplored in the field of neurodegeneration, polyamine dysregulation occurs in numerous disease states, including cancer [78], Parkinson’s disease [79], Multi-System Atrophy [34], and Snyder-Robinson Syndrome [19, 80]. More specifically, during disease states in which tau neuropathology is present, including AD [35–42], dysregulation may contribute to development of further neuropathology and promote disease continuance. Further research remains essential to uncover how tau initiates the polyamine stress response (PSR) and how the PSR feeds back onto the tau phenotype.

Herein, we demonstrate two animal models of tauopathy, with distinct polyamine dysregulation. Directly comparing the two tauopathy models, rTg4510 tau transgenic overexpression and AAV9 Tau ΔD421 viral-mediated overexpression, we observed changes in expression of enzymes involved in polyamine synthesis and polyamine acetylation. We show that spermidine and spermine prevent tau fibrillization but acetylspermidine enhances fibrillization in vitro. In cell culture models, acetylspermidine also promotes tau oligomerization. SSAT ablation in mice reduces acetylputrescine and acetylspermidine and reduces monomeric and high molecular weight tau at certain epitopes but produces some behavioral alterations independent of tau's effects. Lastly, SSAT repression in C3 HeLa cells stably overexpressing tau also reduces monomeric total and phospho-tau at certain epitopes. These data suggest potential interaction in the polyamine pathway and tau biology and warrants further investigation through alternative tau models.

It is worth noting that while increases in putrescine and acetylspermidine were observed in rTg4510 mice, there were no observed changes in any of the other polyamines or acetylated polyamines in response to tau neuropathology. We hypothesize spermidine may act as an intermediary buffer to aid in polyamine homeostasis, due to its relative abundance in comparison to other polyamines and acetylated polyamines. Here, the otherwise increased levels of spermidine may have been circumvented by SSAT-dependent polyamine back-conversion, resulting in increased levels of putrescine and increased levels of acetylspermidine. Support for this notion comes from the decreased levels of ODC, which might have otherwise explained the increase in putrescine, and the observed changes in SRM, SMS, and SMOX that would theoretically result in increased levels of spermidine. In this context, recycling of spermidine via SSAT, to acetylspermidine and putrescine, might serve as a compensatory mechanism in an attempt to rebalance polyamine homeostasis. However, despite the increase in putrescine, the unchanged levels of acetylputrescine remain curious, as this is thought to be under the control of SSAT-dependent catabolism. This also may be due to its detection and overall abundance, as its levels are roughly 5-fold less than acetylspermine and 40-fold less than acetylspermidine, making detection of significant fluctuations more difficult. As for the unchanged levels of acetylspermine, since the levels of spermine were not increased in response to tau neuropathology, which is not surprising given the decrease in SMS and increase in SMOX, we interpret these data as biased SSAT activity directed toward spermidine rebalancing in this model.

These data remain highly important because of a potential feed-forward cycle between tau’s effects and polyamine dysfunction. To the extent that tau initiates a PSR in the brain during tauopathies, the flux in acetylspermidine levels, seen in both tauopathy models presented here, would arguably endorse the tau phenotype. The observed increase in tau fibrillization and oligomerization in response to acetylspermidine supports that notion. In our studies, we measured polyamines and acetylated polyamines in total brain homogenates and were able to detect similar signatures in two models of tau deposition; however, it also remains feasible that polyamines and their byproducts are differentially stored during homeostasis and pathological conditions, which may impact tau aggregation. Normally, acetylated polyamines can be cleared from the cell through SLC3A2 or other transporters [81, 82]; however, it is tempting to speculate that tau may impair clearance as well. Recently, SLC3A2 has been associated with endoplasmic reticulum stress signaling which regulates the unfolded protein response [83]. Importantly, accumulation of acetylated polyamines may not be as inert as previously thought and warrants further investigation during stress paradigms.

Lastly, we demonstrate that by reducing the tau-induced polyamine stress response (tau-PSR), through targeted disruption of SSAT and subsequent reduction in acetylated byproducts, we can subvert some forms of phosphorylation and high molecular weight tau in viral-mediated tau deposition. It is worth noting that the ability of SSAT disruption and the interaction of these specific tau epitopes is likely regionally specific, as highlighted by the reduction in tau pS199/202 the CA3 of the hippocampus measured by IHC but not in the whole hippocampus measured by western blot. Further, models of tauopathy, both viral and transgenic, come with some level of limitation. Here, we show both similarities and differences between rTg4510 and AAV9 Tau ΔD421-treated mice in regard to a tau-PSR. While consistent increases in putrescine and acetylspermidine demonstrate reliability of the tau-PSR, we find some differences in enzymatic dysregulation to be equally intriguing from the possibility of distinct alterations in polyamine signatures across models, which might aid in identifying various forms of tauopathy. In regard to AAV9 Tau ΔD421 as a viral tauopathy model, while inflammation and diverse neuropathology, including phospho-tau epitopes, were produced and the latter being subsequently influenced by SSAT expression, the impact on cognitive performance was significant but only evident in the context of spatial working memory and only as a main effect of treatment. These data suggest that the AAV9 Tau ΔD421 model may be appropriate for biochemical studies and limited for behavioral studies under these conditions. Alternatively, additional injection sites of viral tau (such as the entorhinal cortex), increasing viral incubation, and increasing viral distribution might produce additional and more robust behavioral impairments.

Polyamines support neuronal function and axonal integrity [17–21]; however, the role/function of acetylated polyamines remains elusive. We show for the first time that an acetylated byproduct, acetylspermidine, increases in tauopathies and fails to mimic certain biological effects of polyamines. Polyamines, and the pathway as a whole, are tightly regulated in their biosynthesis, catabolism, and/or transport. Enzymatic activity within the polyamine pathway is regulated at the level of transcription, translation, and protein degradation, involving several feedback loops, and is controlled by polyamine concentrations within the cell. When polyamine metabolism is disrupted, a number of cellular processes are affected, including transcription, translation, gene expression regulation, autophagy, and stress resistance [18]. The notion of a polyamine stress response (PSR) is not a new one [30, 31, 64, 65, 69, 84–87], but has been historically associated with emotional stress seen in psychopathology, including depression [31, 64] and suicidality [66–69, 87–91]. While downregulation of SSAT expression has been observed in the brains of suicide completers [87, 89], some reports show no change in SSAT expression [90], and even an increase in peripheral SSAT expression in suicidal patients [91], leaving the role of SSAT in affective processing and behavioral responses unclear. Interestingly, in the present study, we show, to our knowledge, the first observed behavioral phenotype in the SSAT-/- mice. Although beyond the scope of this study, we report a significant increase in NeuN expression in the hippocampus and cortex in SSAT null mice (Fig. 4a–d), which is also observed in neurological conditions [92, 93].

Physical stressors, such as traumatic brain injury (TBI), can also induce a unique PSR [32]. More specifically, following a lateral controlled cortical impact mode of TBI, SSAT mRNA was increased in both the cortex and the hippocampus of rats. In line with the SSAT-dependent polyamine back-conversion; putrescine and acetylspermidine were also elevated near the site of injury. Intriguingly, TBI is also associated with increased cleavage of tau at aspartate 421 (tau ΔD421) [94], and serves as a risk factor for the development of AD [95, 96]. Here, we present a similar PSR in a viral-mediated model of tauopathy using tau ΔD421 overexpression, in which SSAT disruption prevented the tau-PSR and reduced specific monomeric and high molecular weight phospho-tau species. Taken together, selective modulation of SSAT during disease states, including TBI [96, 97], chronic traumatic encephalopathy (CTE) [98, 99], and Alzheimer’s disease AD [4, 5, 100], may impact tau aggregation.

Polyamines represent an exciting new area of research in tauopathies and AD, serving as natural endogenous inhibitors of tau aggregation. The data presented here provide evidence for a tau-dependent polyamine stress response (tau-PSR) in which pathological tau induces a maladaptive dysregulation in polyamine enzymes which in turn disrupts polyamine metabolism. More specifically, we show that tau promotes a PSR via SSAT, which results in increases acetylated byproducts and thereby may further endorse tau pathogenesis. Support for this notion comes from the targeted disruption or repression of SSAT, subsequent reduction in tau-induced acetylated byproducts, and prevention of certain monomeric and high molecular weight tau phospho-epitopes in vivo and in vitro. Further evidence originates from the increased tau fibrillization and oligomerization following treatment with higher-order acetylated byproducts. However, despite the decrease in ODC and increase in putrescine and acetylspermidine, the present qRT-PCR analysis did not reveal a significant induction of SSAT by AAV9 Tau ΔD421, identifying a possible need for an alternative method for SSAT measurements, such as enzymatic activity quantification. Other methods, such as HPLC [101], immunohistochemistry [32], or GS-MS [69], have been utilized to quantify polyamines and polyamine enzymatic activity and have produced mixed results. Attempts at quantifying SSAT expression by standard western blot, beyond simple absence or presence following siRNA repression, and immunofluorescence have proven inconsistent [32], also identifying a need for improved antibodies against SSAT. Furthermore, previous studies utilizing SSAT-/- mice (referred to as SSAT-ko mice) also found their polyamine profile to be less defining, primarily due to inherently low enzyme activities and polyamine content. More specifically, no significant change in liver and white adipose tissue SSAT enzyme activity was detected in SSAT-ko mice, citing the presence of nonspecific acetylating enzymes as a possible culprit [102]. Additionally, using HPLC, the previous report found non-detectible levels of acetylspermidine and did not report levels of acetylputrescine or acetylspermine [45]. While we were able to quantify all polyamines and acetylated byproducts using LC-MS/MS, the polyamine profile of SSAT-/- mice remains unclear and warrants further consideration.

Lastly, while SSAT disruption demonstrated as an effective therapeutic target for some aspects of the tau phenotype, namely the tau-PSR and accumulation of some phospho-species in vivo and in vitro, secondary effects on behavioral performance demonstrate that targeting SSAT via conventional whole-body disruption may lack specificity. Current available antagonists for SSAT also present with a multitude of side effects and lack specificity for SSAT alone; therefore, future studies should investigate conditional genetic knockdown of SSAT, as an alternative method of targeting the tau-PSR, as well as an attempt to elucidate the exact mechanism(s) by which polyamines exert their effects on tau pathology.

## Conclusion

In conclusion, we demonstrate that tau induces a polyamine stress response (tau-PSR) in two models harboring tau neuropathology. Further, we show the relationship between tau and polyamine dysregulation as being maladaptive and direct, in that some SSAT-dependent acetylated polyamine byproducts promote tau fibrillization and oligomerization. Targeted disruption of SSAT is capable of preventing certain components of the tau-PSR and the accumulation of specific phospho-tau epitopes and high molecular weight multimers but also produces alterations in behavioral performance independent of tau. These data represent a novel but complex paradigm linking tau pathology and polyamine dysfunction.

## Additional file

Additional file 1: **Supplemental figure 1**. Polyamine quantification (putrescine, spermidine, acetylputrescine, acetylspermidine) of brain homogenates in response to 4-month incubation of either AAV9 Empty Capsid (EC) or AAV9 Tau ΔD421 in 15-month old nTg and SSAT-/- mice (n=9-11). **a** Putrescine:Simple main effects analysis showed that SSAT disruption significantly reduced putrescine (F(1,35) = 332.618, p = .000). Further, within each genotype, pairwise comparison revealed a trend in difference in putrescine between only the nTg AAV9 Empty Capsid and nTg AAV9 Tau ΔD421 groups (p = .061). No main effect of AAV9 Tau ΔD421 (F(1,35) = 1.698, p = .201) or interaction of factors (F(1,35) = 1.960, p = .170) was detected on putrescine. **b** Acetylputrescine: Simple main effects analysis showed that SSAT disruption significantly reduced acetylputrescine (F(1,37) = 57.551, p = .000). No main effect of AAV9 Tau ΔD421 (F(1,37) = .075, p = .785) or interaction of factors (F(1,37) = .008, p =.928) was detected on acetylputrescine. **c** Spermidine: Simple main effects analysis showed that SSAT disruption significantly reduced spermidine (F(1,37) = 4.736, p = .036). No main effect of AAV9 Tau ΔD421 (F(1,37) = .001, p = .970) or interaction of factors (F(1,37) = 2.009, p =.165) was detected on spermidine. **d** Acetylspermidine: Simple main effects of genotype (F(1,37) = 27.723, p = .000), AAV9 Tau ΔD421 (F(1,37) = 11.661, p = .002) and interaction of factors (F(1, 37) = 6.539, p = .015) was detected on acetylspermidine. **e** Spermine: No main effect of genotype (F(1,35) = 2.012, p = .165), AAV9 Tau ΔD421 (F(1,35) = .236, p = .630), or interaction of factors (F(1,35) = .296, p = .590) was detected on Spermine. **f** Acetylspermine: Simple main effects analysis showed that SSAT disruption significantly increased acetylspermine (F(1, 38) = 9.360, p = .004). No main effect of AAV9 Tau ΔD421 (F(1,38) = .004, p = .949) or interaction of factors (F(1, 38) = 2.439, p = .127) was detected on acetylspermine. **g** SSAT mRNA: mRNA expression of SSAT in response to 4-month incubation of either AAV9 Empty Capsid (EC) or AAV9 Tau ΔD421 in 15-month old nTg and SSAT-/- mice (n=3-5).Simple main effects showed that SSAT disruption significantly reduced SSAT expression, normalized to GAPDH, (F(1, 12) = 20.660, p = .001. No effect of AAV9 Tau ΔD421 (F(1,12) = .687, p = .423) or interaction of factors (F(1,12) = .694, p = .421) was detected on SSAT expression. 2x2 Factorial analysis of variance (ANOVA), followed by pair-wise comparisons using Fishers PLSD. *p <.05; asterisk indicate the main effect of treatment and main effect of genotype, ampersands indicate the interaction of genotype and treatment, number signs indicate pairwise comparison within genotype. Data is represented by means ± S.E.M. (TIF 747 kb)

## Data Availability

The datasets used and/or analyzed during the current study are available from the corresponding author on reasonable request.

## Abbreviations

A.U: Arbitrary units
AAV9 Tau ΔD421; virus, tau ΔD421; antibody: Caspase 3-cleaved tau truncated at aspartate 421
AD: Alzheimer’s disease
AUC: Area under the curve
BiFC: Bimolecular fluorescence complementation
CaMKII-α: Calcium/calmodulin-dependent protein kinase type II alpha chain
CBD: Corticobasal degeneration
DENSPM/DSPM: N1, N11-diethylnorspermine
DPBS: Dulbecco’s phosphate-buffered saline
EPM: Elevated plus maze
IHC: Immunohistochemistry
LC-MS/MS: Liquid chromatography tandem mass spectrometry
LTP: Long-term potentiation
MB: Marble burying task
N2a-ssGT: Murine neuroblastoma monoclonal superfolder split GFP-tau cell line
NFTs: Neurofibrillary tangles
Nrf-2: NF-E2 released factor 2
OAZ1: Ornithine decarboxylase antizyme 1
ODC: Ornithine decarboxylase
ODC: Ornithine decarboxylase
PAOX: Polyamine oxidase
PBS: Phosphate-buffered saline
PHFs: Paired helical filaments
PiD: Pick’s disease
PMF1: Polyamine modulated factor 1
PMF1: Polyamine modulated factor 1
PMFBP1: Polyamine-modulated factor binding protein 1
PMFBP1: Polyamine-modulated factor 1 binding protein 1
PSP: Progressive supranuclear palsy
PSR: Polyamine stress response
rAAV: Recombinant adeno-associated virus
RAWM: Radial arm water maze
RFU: Relative fluorescence unit
SMOX: Spermine oxidase
SMS: Spermidine synthase
SMS: Spermine synthase
SRM: Spermidine synthase
SSAT: Spermidine/spermine-*N*^1^-acetyltransferase
Tau-PSR: Tau-induced polyamine stress response
TBI: Traumatic brain injury
ThT: Thioflavin T assay
WB: Western blotting
WT: Wild type
