# Supplementary figures and images for: Spermidine/spermine-N1-acetyltransferase ablation impacts tauopathy-induced polyamine stress response
Source: Alzheimers Res Ther. 2019 Jun 29;11:58. doi: 10.1186/s13195-019-0507-y (PMC6599347; doi:10.1186/s13195-019-0507-y)

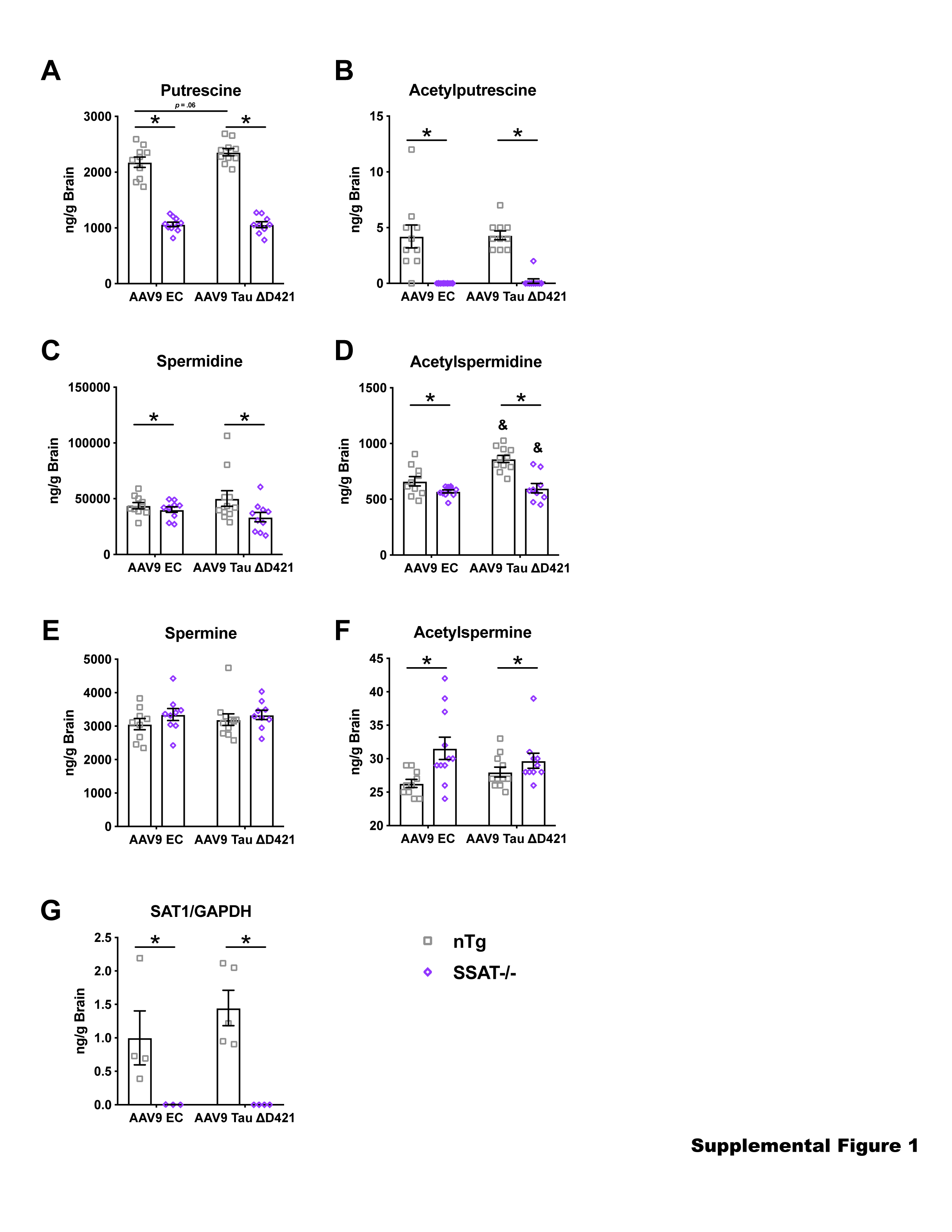

Supplement: Supplementary file 1 — Supplemental figure 1. Polyamine quantification (putrescine, spermidine, acetylputrescine, acetylspermidine) of brain homogenates in response to 4-month incubation of either AAV9 Empty Capsid (EC) or AAV9 Tau ΔD421 in 15-month old nTg and SSAT-/- mice (n=9-11). a Putrescine:Simple main effects analysis showed that SSAT disruption significantly reduced putrescine (F(1,35) = 332.618, p = .000). Further, within each genotype, pairwise comparison revealed a trend in difference in putrescine between only the nTg AAV9 Empty Capsid and nTg AAV9 Tau ΔD421 groups (p = .061). No main effect of AAV9 Tau ΔD421 (F(1,35) = 1.698, p = .201) or interaction of factors (F(1,35) = 1.960, p = .170) was detected on putrescine. b Acetylputrescine: Simple main effects analysis showed that SSAT disruption significantly reduced acetylputrescine (F(1,37) = 57.551, p = .000). No main effect of AAV9 Tau ΔD421 (F(1,37) = .075, p = .785) or interaction of factors (F(1,37) = .008, p =.928) was detected on acetylputrescine. c Spermidine: Simple main effects analysis showed that SSAT disruption significantly reduced spermidine (F(1,37) = 4.736, p = .036). No main effect of AAV9 Tau ΔD421 (F(1,37) = .001, p = .970) or interaction of factors (F(1,37) = 2.009, p =.165) was detected on spermidine. d Acetylspermidine: Simple main effects of genotype (F(1,37) = 27.723, p = .000), AAV9 Tau ΔD421 (F(1,37) = 11.661, p = .002) and interaction of factors (F(1, 37) = 6.539, p = .015) was detected on acetylspermidine. e Spermine: No main effect of genotype (F(1,35) = 2.012, p = .165), AAV9 Tau ΔD421 (F(1,35) = .236, p = .630), or interaction of factors (F(1,35) = .296, p = .590) was detected on Spermine. f Acetylspermine: Simple main effects analysis showed that SSAT disruption significantly increased acetylspermine (F(1, 38) = 9.360, p = .004). No main effect of AAV9 Tau ΔD421 (F(1,38) = .004, p = .949) or interaction of factors (F(1, 38) = 2.439, p = .127) was detected on acetylspermine. g SSAT mRNA: mRNA exp [file 13195_2019_507_MOESM1_ESM.tif]
